# Controlled release of hydrogen isotope compounds and tunneling effect in the heterogeneously-catalyzed formic acid dehydrogenation

**DOI:** 10.1038/s41467-019-12018-7

**Published:** 2019-09-25

**Authors:** Kohsuke Mori, Yuya Futamura, Shinya Masuda, Hisayoshi Kobayashi, Hiromi Yamashita

**Affiliations:** 1 Division of Materials and Manufacturing Science, Graduate School of Engineering, Osaka University, 2-1 Yamada-oka, Suita, Osaka 565-0871 Japan; 2 Elements Strategy Initiative for Catalysts Batteries ESICB, Kyoto University, Katsura, Kyoto 615-8520 Japan; 3 Kyoto Institute of Technology, Matsugasaki, Sakyo-ku, Kyoto 606-8585 Japan

**Keywords:** Catalytic mechanisms, Heterogeneous catalysis, Synthesis and processing

## Abstract

The hydrogen isotope deuterium is widely used in the synthesis of isotopically-labeled compounds and in the fabrication of semiconductors and optical fibers. However, the facile production of deuterium gas (D_2_) and hydrogen deuteride (HD) in a controlled manner is a challenging task, and rational heterogeneously-catalyzed protocols are still lacking. Herein, we demonstrate the selective production of hydrogen isotope compounds from a combination of formic acid and D_2_O, through cooperative action by a PdAg nanocatalyst on a silica substrate whose surface is modified with amine groups. In this process, D_2_ is predominantly evolved by the assist of weakly basic amine moieties, while nanocatalyst particles in the vicinity of strongly basic amine groups promote the preferential formation of HD. Kinetic data and calculations based on semi-classically corrected transition state theory coupled with density functional theory suggest that quantum tunneling dominates the hydrogen/deuterium exchange reaction over the metallic PdAg surfaces.

## Abstract (web summary)

Hydrogen isotope compounds are useful in fine chemical production, but the natural abundance of these gases is low and the techniques used for producing D_2_ are expensive and energy intensive. Here, the authors develop a nanocatalyst that promotes selective production of D_2_ and HD from the inexpensive starting material formic acid in the presence of D_2_O.

## Introduction

Hydrogen isotopes, such as deuterium gas (D_2_) and hydrogen deuteride (HD), are minor components of naturally occurring molecular hydrogen, and have applications as fine chemicals owing to their unique physical, quantum, and nuclear properties^1^. In particular, D_2_ is often employed for the synthesis of a diverse array of deuterium-labeled compounds, which have been recognized as indispensable research tools in the life, environmental, and material sciences because of their non-radioactive and stable characteristics^2–6^. Unfortunately, commercially available D_2_ gas is quite expensive, since it is produced by the electrolysis of D_2_O with the investment of large amounts of energy. The reactions of various metals, including Na, Fe, and Mg, with D_2_O have also been traditionally employed to generate this gas on a laboratory scale, but these reagents have serious drawbacks in that they are toxic, produce large amounts of waste, and require high reaction temperatures^7^.

A number of metal-catalyzed H/D exchange reactions between H_2_ and D_2_O, especially those based on Ir^8^, Rh^9,10^, and Ru^11,12^, have been developed based on the investigation of hydrogenase enzymes^13^. Sajiki and co-workers^14^ explored Pd/C-catalyzed D_2_ generation via H/D exchange reactions using a H_2_/D_2_O combination, and applied the D_2_ that was generated in situ to the synthesis of D-labeled compounds. More recently, Fujita and co-workers^15^ developed a catalytic method to produce D_2_ from deuterated methanol (CD_3_OD) and D_2_O using an Ir complex bearing functional bipyridonate ligands in the presence of sodium hydroxide.

Formic acid (HCOOH, FA) has been the subject of significant research effort because it is one of the most promising hydrogen storage materials, due to its low toxicity, nonflammable liquid state at room temperature, and high hydrogen content (43.8 g H_2_ kg^−1^ or 52 g H_2_ L^−1^)^16–18^. Systems involving FA can also allow economical CO_2_-mediated hydrogen storage energy cycling via the regeneration of FA through the hydrogenation of CO_2_^19–22^. Driven by these intrinsic advantages, numerous investigations have been conducted with the aim of identifying highly active catalysts for the dehydrogenation of FA, which has resulted in relatively sophisticated systems intended for industrial applications^23,24^. H_2_ can be controllably released through the thermodynamically favored dehydrogenation of FA (HCOOH → H_2_ + CO_2_, Δ*G* = −48.4 kJ mol^−1^) in the presence of suitable catalysts. However, in such systems, complete suppression of the side reaction (HCOOH → CO + H_2_O, Δ*G* = −28.5 kJ mol^−1^) is required, because of the potential for catalyst inhibition by CO poisoning.

Interestingly, hydrogen isotope compounds have been evolved via the H/D exchange reaction during the metal-catalyzed dehydrogenation of FA in D_2_O. In an early study, Puddephatt and co-workers^25^ demonstrated that a mixture of H_2_, HD, and D_2_ gases in a molar ratio of 1:2:1 can be generated by reactions between HCOOD and D_2_O or between DCOOH and H_2_O, catalyzed by a binuclear Ru complex. Subsequently, the distribution of D during the decomposition of deuterated FA analogs (i.e., HCOOD, DCOOH, and DCOOD) in D_2_O in the presence of [Rh^III^(Cp*)(bpy)(H_2_O)]^2+^ and the heterodinuclear iridium–ruthenium complex [Ir^III^(Cp*)(H_2_O)(bpm)Ru^II^(bpy)_2_](SO_4_)_2_ (Cp* = pentamethylcyclopentadienyl, bpm = 2,2′-bipyrimidine, bpy = 2,2′-bipyridine) was explored by Fukuzumi et al.^26,27^. A convenient means of producing D_2_ and various deuterated compounds using a half-sandwich Ir complex with 4,4′-dihydroxy-2,2′-bipyridine ligands was also demonstrated by Himeda and co-workers^28^. Although some progress has been achieved in conjunction with the use of transition metal complexes, these processes often require high catalyst concentrations and expensive deuterated FA to obtain reasonable levels of activity and selectivity. In addition, the use of homogeneous catalysts on an industrial scale is extremely challenging due to the difficulties involved in recovering the precious metals and ligands from the reaction mixture. For these reasons, systems based on heterogeneous catalysts in association with undeuterated FA and D_2_O would be highly beneficial, but such processes have not yet been researched, in spite of the obvious practical advantages.

Our group has previously reported that the presence of basic functional groups on support materials in the vicinity of active Pd-based alloy nanoparticles (NPs) promotes the production of high-purity H_2_ during the dehydrogenation of FA^29–34^. Herein, we describe a straightforward method for the production of hydrogen isotope compounds from a combination of FA and D_2_O, using a PdAg nanocatalyst on a substrate that has been surface modified with amine groups. This work also demonstrates that the selectivity of this process can be tuned by varying the basicity of the amine moieties. The H/D exchange reactions between FA and D_2_O is found to involve a quantum tunneling effect, as evidenced by the large kinetic isotope effect (KIE) value, and based on semi-classically corrected transition state theory (SC-TST) coupled with density functional theory (DFT) calculations.

## Results

### Structural Characterization

**Fig. 1:**
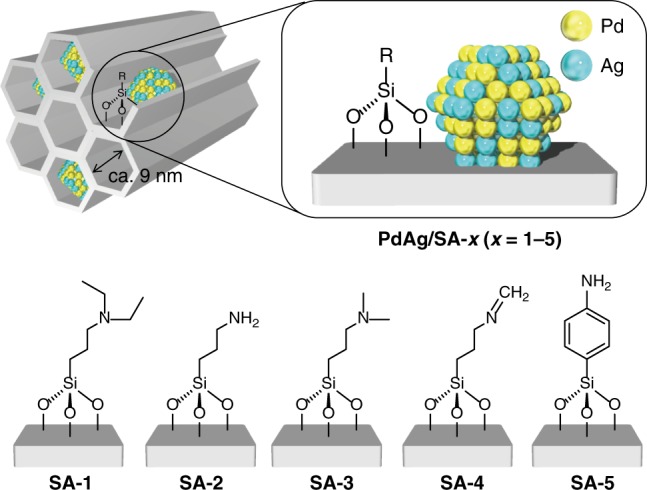
Schematic illustrations of catalysts. The PdAg nanoparticles were deposited on the surface of SBA-15 mesoporous silica modified with silane-coupling reagents containing different amine groups (termed SA-*x*, where *x* = 1–5)

Various amine-grafted mesoporous silica (SBA-15) substrates (termed SBA-15-Amine-x (SA-*x*), where *x* *=* 1–5) were synthesized using a surface modification technique. This silica possesses relatively large hexagonal channels ~9 nm in diameter. The surface modification was performed using a post-synthesis method in association with silane-coupling reagents containing different amine groups, as shown in Fig. 1. The SA-4 specimen, having Schiff base functional groups (-N = CH_2_), was also synthesized by the further modification of SA-2 with formaldehyde. These substrates were impregnated with Pd and Ag (1 wt% Pd; Pd:Ag molar ratio = 1:1) simply by immersion in aqueous solutions of Pd(NH_3_)_4_Cl_2_ and AgNO_3_ followed by reduction with NaBH_4_. The resulting catalysts are denoted PdAg/SA-*x* (*x* *=* 1–5) herein. The concentration of grafted amine functionalities in all samples was determined to be ~0.3 mmol g^−1^ substrate using thermogravimetric (TG) analysis. The presence of the amine groups was confirmed by the analysis of each specimen by Fourier transform-infrared (FT-IR) spectroscopy (Supplementary Fig. 1) and N 1*s* X-ray photoelectron spectroscopy (XPS) (Supplementary Fig. 2). The long-range ordering of hexagonally packed mesoporous structures in these materials was also demonstrated on the basis of low-angle X-ray diffraction (XRD) patterns (Supplementary Fig. 3).

The textural properties of the PdAg/SA-*x* as well as PdAg/S made with unmodified silica are summarized in Table S1. All samples generated N_2_ adsorption/desorption isotherms having characteristic type IV curves with sharp capillary condensation steps and H1-type hysteresis loops. These features indicate highly ordered and well-preserved cylindrical mesopores (Supplementary Fig. 4). The Brunauer–Emmett–Teller (BET) surface areas (*S*_BET_), pore diameters (*D*_pore_) and pore volumes (*V*_p_) for the members of the PdAg/SA-*x* series were all found to be slightly lower than those for the unmodified PdAg/S. During surface modification, the silane-coupling reagents were able to readily diffuse through the channels, which ensured homogeneous grafting, because the estimated molecular sizes of these reagents (up to 1 nm) were significantly smaller than the diameters of the channels.

Supplementary Figure 5 presents high-angle annular dark-field scanning transmission electron microscopy (HAADF-STEM) images together with size distribution plots for the PdAg/S and PdAg/SA-*x* materials. The PdAg NPs on the PdAg/SA-*x* were evidently well dispersed and had a narrow size distribution, with average diameters in the range of 4.5–7.3 nm. These values are smaller than the 7.5 nm NP size on the unmodified PdAg/S, and so amine functionalization evidently resulted in the formation of smaller NPs. This result indicates that variations in the basicity of the grafted amine groups may affect both the formation of Pd metal nuclei and the consecutive growth, ultimately producing different NP sizes.

The shapes of the Pd K-edge X-ray absorption near edge structure (XANES) spectra generated by the PdAg/SA-*x* and unmodified PdAg/S differ from that seen in the PdO spectrum, but resemble the spectrum of Pd foil (Supplementary Fig. 6A). The FT-extended X-ray absorption fine structure (FT-EXAFS) spectra of PdAg/SA-*x* and PdAg/S exhibit a single intense peak associated with Pd−Pd bonds that suggests the presence of metallic Pd (Supplementary Fig. 6B). However, the Pd–Pd bond lengths for these materials were slightly longer than that for Pd foil, indicating Pd−Ag bonds. The Ag K-edge XANES spectra obtained from the PdAg/SA-*x* and PdAg/S are similar to that of Ag foil, and the FT-EXAFS spectra of these specimens exhibit a single sharp peak ascribed to contiguous Ag–Ag bonds with lengths of ~2.6–2.8 Å (Supplementary Fig. 7). This peak is shifted to a slightly shorter interatomic distance compared with that for pure Ag foil. These results present clear evidence for the formation of PdAg alloy NPs. Additionally, the Pd 3*d* peaks in the XPS spectra produced by the PdAg/SA-*x* are slightly shifted to higher binding energies compared to those for the unmodified sample (Supplementary Fig. 8A). A similar shift of the Ag 3*d* peaks in the XPS spectra is also evident after modification (Supplementary Fig. 8B). Our experimental results suggest that surface amine functional groups were present at the peripheries of the loaded PdAg NPs to alter their electronic state. The alternation of the electronic state of the supported NPs by the organic modifier of the support materials have also been reported previously^33^.

### Controllable release of hydrogen isotope compounds

**Fig. 2:**
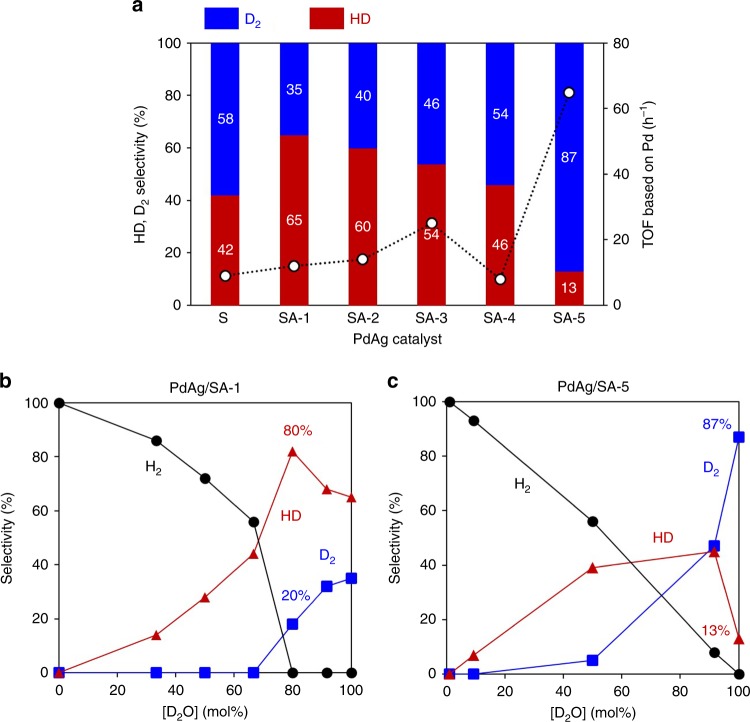
Selectivity and TOF for the dehydrogenation of FA. **a** Effect of catalysts using PdAg/S and PdAg/SA-*x* (*x* = 1–5); **b** effect of D_2_O mol% in solution using PdAg/SA-1. **c** Effect of D_2_O mol% in solution using PdAg/SA-5

Utilizing the prepared catalysts having different amine functional groups, the dehydrogenation reaction was conducted in D_2_O solutions containing 5 M HCOOH:HCOONa (9:1) at 343 K for 30 min. The selectivities for the resulting hydrogen isotope compounds (HD and D_2_) are summarized in Fig. 2a, together with the turnover frequency (TOF) values based on the amount of Pd. No H_2_ formation was observed, and the molar ratios of total hydrogen isotope compounds to CO_2_ generated during the course of the reaction were close to 1 for all samples. The use of bare SA-5 without PdAg NPs does not show any activity. It should be noted that the present catalytic system limits the undesirable formation of CO to less than the detection limit of the gas chromatography (GC) system (<2 ppm). As expected, we succeeded in the controlled synthesis of these hydrogen isotope compounds by changing the surface grafted amine groups. As an example, the PdAg/SA-1 (having the strongly basic −NEt_2_ groups) preferentially generated HD with 65% selectivity, while D_2_ formation was predominant with 87% selectivity when using the PdAg/SA-5, which was modified with the weakly basic –PhNH_2_ groups. The D_2_ and HD selectivity appeared to be independent of the yield level and remained unchanged during the reaction, suggesting that the H–D exchange reaction with the produced hydrogen isotope compounds did not take place (Supplementary Fig. 9). In addition, the catalytic activity was also greatly affected by the properties of the supports. As an example, the TOF value obtained with PdAg/SA-5 was 65 h^−1^, which was seven times that observed using the PdAg/SA-1.

The H_2_O/D_2_O molar ratio in the reaction solution also significantly affected selectivity. Fig. 2b, c plots the selectivity for various hydrogen isotope compounds versus the D_2_O mol%. In the case of the PdAg/SA-1, HD selectivity increased with increasing D_2_O concentration, accompanied by a decrease in H_2_, up to a D_2_O concentration of 67 mol%, with no D_2_ formation observed. The maximum HD selectivity of 80% was attained at 80 mol% D_2_O without the formation of H_2_, after which the HD selectivity decreased. In the reaction with PdAg/SA-5, the HD selectivity increased along with the D_2_O mol%, while the D_2_ selectivity was drastically enhanced at D_2_O levels above 50 mol%. The highest D_2_ selectivity was obtained at 100 mol% D_2_O, with 87% selectivity. In contrast, the type of amine functional group, PdAg NP loading, reaction temperature, and ratio of FA to sodium formate did not show significant effect on selectivity (Supplementary Figs. 10–13), demonstrating that these parameters are minor factors in determining the selectivity.

The recyclability is a crucial point to consider when heterogeneous catalysts are employed for industrial applications. After the reaction, the PdAg/SA-*x* (*x* = 1 and 5) catalysts were easily separated from the reaction mixture and could be reused with retention of its activity and selectivity; preferential formation of hydrogen isotope compounds could be attained for at least five recycling experiments (Supplementary Fig. 14). More interestingly, the present catalyst provides a simple and efficient heterogeneous catalytic system for the in situ deuteration of organic compounds (Table 1 and Supplementary Fig. 15). As an example, the reaction of diphenylacetylene proceeded smoothly using PdAg/SA-5 and 5 M HCOOH:HCOONa (9:1) solution in D_2_O, giving the deuterated product with 96% deuterated content in 99% chemical yield. The expected deuterated products were also obtained with excellent deuterated contents via addition reactions of deuterium to carbon–carbon double bonds and deuterium substitution reactions.

**Table 1:**
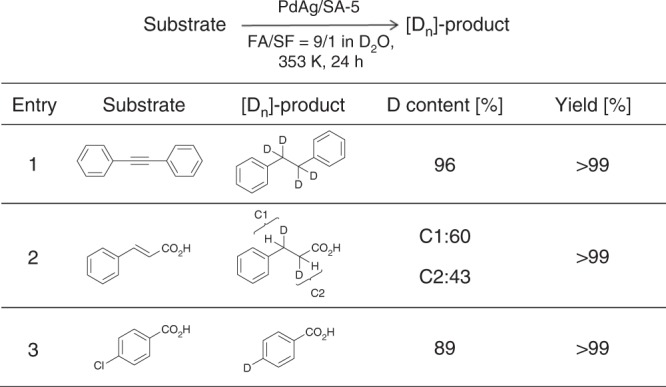
Results of catalytic deuteration

**Fig. 3:**
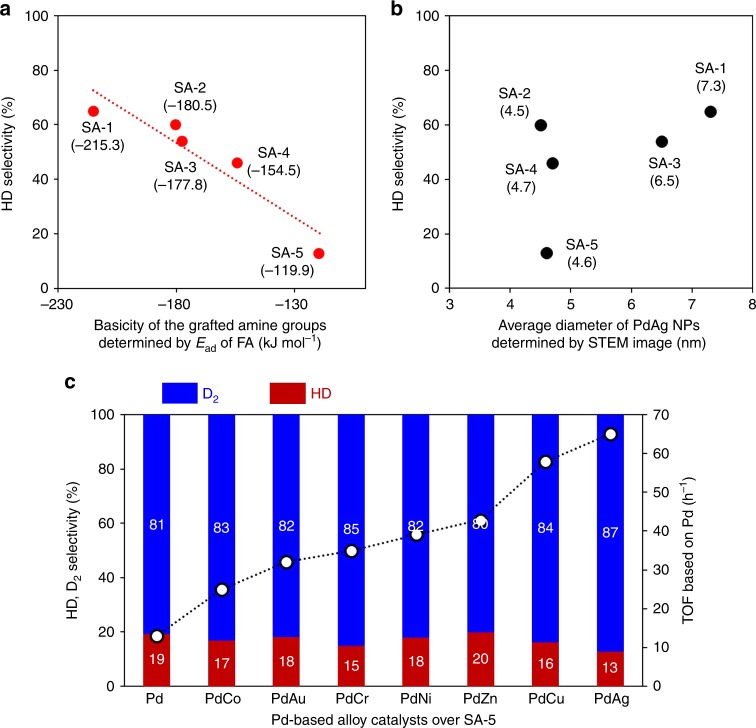
Key factor for determining selectivity. **a** Correlations between HD selectivity and basicity of the amine groups determined by the adsorption energy (*E*_ad_) of FA; **b** correlations between HD selectivity and the average diameter of PdAg NPs as determined from STEM; **c** selectivity and TOF values using Pd-based alloy NPs supported on SA-5

The observed variations in selectivity for hydrogen isotope compounds were assessed by determining the adsorption energy (*E*_ad_) of FA based on DFT calculations for each SA-*x*. This was done because such values reflect the basicity of the amine functional groups, since the adsorption involves formation of an acid–base pair via N–H···O hydrogen bonding^32^. As an example, the *E*_ad_ over SA-1 with −N(C_2_H_5_)_2_ groups was found to be −215 kJ mol^−1^, which was substantially stronger than the value of −120 kJ mol^−1^ over SA-5, having –PhNH_2_ groups. It is worth noting that a good correlation between the HD selectivity and *E*_ad_ was observed. Specifically, the HD selectivity increased with increases in *E*_ad_, as shown in Fig. 3a, suggesting that the basicity of the amine functional groups plays a crucial role in determining selectivity.

We suspected that differences in the sizes of the PdAg NPs also affected selectivity. However, there was no significant correlation between selectivity and the average diameter of PdAg NPs on the SA-*x* series of samples, based on HAADF-STEM analysis (Fig. 3b). Unfortunately, this investigation was unable to accurately decouple the concomitant effect of the basicity of the various amine functional groups from that of the NP size. In order to further examine the effect of NP size on selectivity, a series of colloidal poly(vinylpyrrolidone)-capped Pd NPs were first synthesized under several different conditions and then deposited on the SA-5^35^. The TEM images and size distribution plots shown in Supplementary Fig. 16 demonstrate the fabrication of size-controlled Pd/SA-5 specimens with particle sizes ranging from 2.8 to 16 nm. These materials were subsequently applied to the dehydrogenation of FA in D_2_O. As expected, the D_2_ selectivity was found to remain almost constant at ~90% regardless of the particle size, although a volcano-type variation in activity based on the particle size was observed, with a maximum TOF at 3.9 nm (Supplementary Fig. 17).

In addition, Pd-based alloy NPs supported on SA-5 were synthesized, using Au, Co, Cr, Ni, Zn, or Cu precursors as the second metal and employing the same impregnation method followed by reduction with NaBH_4_. Notably, these variations in the metal component did not greatly affect the D_2_ selectivity (Fig. 3c), although the activity varied significantly. This effect can presumably be ascribed to the synergic effect between the Pd and the second metal, as has been reported previously^29,30,36–38^. These results suggest that the selectivity for the production of hydrogen isotope compounds is governed by the grafted amine groups in the periphery of each active center, and not by the inherent ability of the metal NPs themselves.

**Fig. 4:**
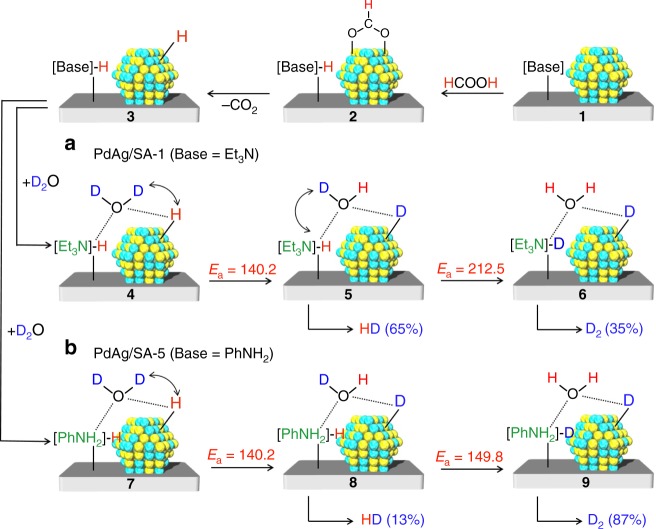
A proposed reaction mechanism in D_2_O. Pathway (a) represents the selective production of HD using PdAg/SA-1 and pathway (b) represents the selective production of D_2_ using PdAg/SA-5. The values above the arrows are activation energy (*E*_a_) (kJ mol^−1^) for each elementary step

Upon consideration of the above results as well as the theoretical studies, we determined a possible reaction pathway leading to the selective formation of hydrogen isotope compounds from the dehydrogenation of FA in D_2_O, as illustrated in Fig. 4. In this process, regardless of the basicity of the amine groups, the reaction of HCOOH with 1 is promoted by the amine groups in the periphery of the PdAg NPs as a result of the acid–base interaction, affording a Pd formate species along with a proton-scavenged amine group (2). The Pd formate species, for which the *trans*-AgO-PdH(O)-bridged configuration is the lowest energy structure, subsequently undergoes isomerization followed by β-hydride elimination to release CO_2_ and a Pd hydride species (3). Intermediate 3 undergoes H–D exchange reactions in the presence of D_2_O at both the Pd and basic amine sites, but to differing extents according to the basicity of the amine groups. In the case of PdAg/SA-1, which has strongly basic −NEt_2_ groups, four preferentially undergoes an H–D exchange reaction at the Pd site with a barrier of *E*_a_ = 140.2 kJ mol^−1^ to afford 5. Subsequently, an H–D exchange reaction proceeds at the basic amine site with a barrier of 212.5 kJ mol^−1^ to produce 6. The intermediates 5 and 6 release HD and D_2_, respectively. Thus, the substantially higher activation energy for the formation of 6 compared to that for 5 contradict the occurrence of the reversed reaction, which also explains the preferential formation of HD. In contrast, the activation energies for the formations of 8 and 9 from 7 via the H–D exchange reaction at Pd and weakly basic −PhNH_2_ groups in the case of PdAg/SA-5 were calculated to be 140.2 and 149.8 kJ mol^−1^, respectively. From an energetic point of view, the H–D exchange reaction would therefore be expected to occur simultaneously at both sites, leading to the preferential formation of intermediate 9 and ultimately favoring D_2_ generation. The *E*_a_ for the H–D exchange reaction at the strongly basic −N(C_2_H_5_)_2_ site is substantially larger than that at the weakly basic −PhNH_2_ site, which are reflected by the basicity determined by the *E*_ad_ of FA in Fig. 3a; the *E*_a_ increased with increasing the *E*_ad_ of FA. It can therefore be concluded that the selectivity for hydrogen isotope compounds is determined by the basicity of the surface amine groups, which ultimately influence on the H–D exchange reaction at the basic sites in the peripheries of the active centers. In addition, the preliminary DFT calculations determined the *E*_a_ for the release of hydrogen from 5 or 6 and 8 or 9 were 52.7 and 13.0 kJ mol^−1^, respectively, indicating rate-determining step is the H–D exchange reaction at the basic amine site with D_2_O. These results also suggest that hydrogen release is accelerated in the presence of weakly basic amines, which accounts for the higher TOF obtained from PdAg/SA-5 as compared to that for PdAg/SA-1, as shown in Fig. 2a.

**Fig. 5:**
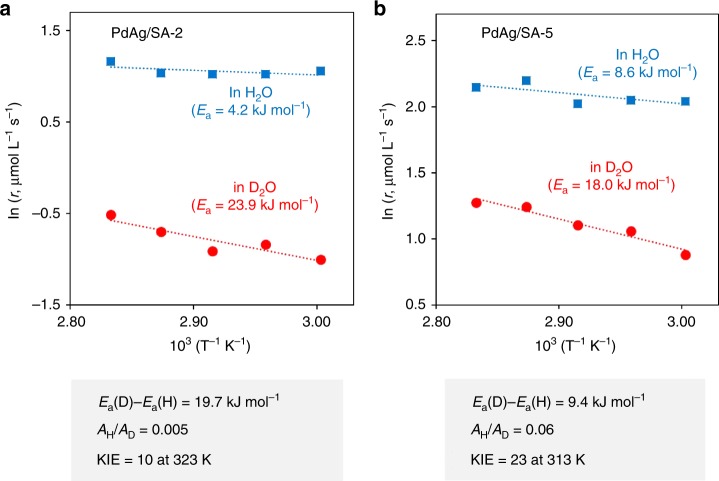
Arrhenius plots in H_2_O and D_2_O. The obtained Arrhenius parameters were represented for **a** PdAg/SA-2 and **b** PdAg/SA-5

To improve our understanding of this mechanism, H/D KIE values were examined in trials using H_2_O and D_2_O. Intriguingly, large KIE values of 10 at 323 K and 23 at 313 K were obtained when using PdAg/SA-2 and PdAg/SA-5, respectively. Abnormally large KIE values at room temperature or higher generally indicate the participation of a quantum tunneling effect in a proton transfer reaction, and this can be unambiguously confirmed from the temperature dependence of KIE. Specifically, values for the Arrhenius parameters *E*_a_(D)–*E*_a_(H), *A*_H_/*A*_D_, and KIE of >5 kJ mol^−1^, ≪1, and >9 at 298 K provide evidence for the involvement of a quantum tunneling effect^27,39^. The Arrhenius plots for H_2_O and D_2_O obtained in the present study are provided in Fig. 5. The resulting *E*_a_(D)–*E*_a_(H) and *A*_H_/*A*_D_ values were 19.7 kJ mol^−1^ and 0.005 for PdAg/SA-2 and 9.4 kJ mol^−1^ and 0.06 for PdAg/SA-5, respectively. Our experimental data therefore meet the above requirements, and indicate the contribution of a quantum tunneling effect to the present catalytic cycle.

### Tunneling effect calculations

Quantum mechanical tunneling of atoms, which originates from wave-particle duality, has been observed not only in chemical reactions, but also in many other fields of chemistry including astrochemistry and biochemistry^40–42^. In particular, the tunneling of hydrogen species such as protons, hydrogen atoms and hydride ions is an important aspect of acid–base, transfer and proton-coupled electron transfer reactions^43–48^. As an example, hydrogen atoms have been frequently observed to tunnel through various metal surfaces, such as Cu(001)^49^, Ru(0001)^50^ and Ni(100)^51^, and the motion of hydrogen atoms is known to be enhanced by tunneling at low temperatures. Proton hopping between oxygen atoms on the Brønsted acidic sites of zeolites and heteropolyacids has also been found to be associated with tunneling pathways^52,53^. In addition, it has been demonstrated that reactions based on heterogeneous catalysis, such as CO oxidation and NH_3_ formation on metal surfaces, are also affected by tunneling^54–58^.

Atom tunneling can be experimentally demonstrated by constructing a non-linear Arrhenius plot based on data acquired at low temperatures, where the rate constant is independent of temperature, or (as described above) by unusual KIE values. However, the effect of tunneling on reaction rates can also be directly confirmed by theoretical calculations^59–62^. In an effort to theoretically demonstrate the participation of tunneling pathways in the present heterogeneously-catalyzed dehydrogenation of FA using PdAg NPs in the vicinity of amine groups, we adopted the semi-classically corrected TST (SC-TST) developed by Fermann and Auerbach^63^, together with a modification by Bhatia and Sholl^64,65^. A tunneling crossover temperature, *T*_c_, was calculated using the equations

1 $$T_{\rm{c}} = \frac{{h\nu _{{\rm{Im}}}^\dagger {\mathrm{\Delta }}E_{{\rm{ZP}}}/k_{\rm{B}}}}{{2\pi {\mathrm{\Delta }}E_{{\rm{ZP}}} - h\nu _{\rm{Im}}^\dagger {\mathrm{ln}}{2}}},$$

2 $${\mathrm{\Delta }}E_{{\rm{ZP}}} = \left( {E^\dagger + \mathop {\sum }\limits_{j = 1}^{N - 1} \frac{{h\nu _j^\dagger }}{2}} \right) - \left({E_{\rm{A}} + \mathop {\sum }\limits_{i = 1}^N \frac{{h\nu _i}}{2}} \right),$$

where Δ*E*_ZP_ is the activation energy barrier (i.e., the difference in energies between the TS and the local minimum, including the SCF energy (*E*^†^ or *E*_A_), and the zero-point energy, as defined in Eq. (2)). *N* is the number of degrees of freedom for the system, *h* and *k*_B_ are the Planck and Boltzmann constants, *v*_l_ is the harmonic frequency for the local minimum, and *v*^†^_Im_ and *v*_*j*_ are those for the TS (the former being an imaginary frequency).

Within the SC-TST framework, the reaction rate constant at temperature *T* is given by Eq. (3), using the TST rate constant, *k*^TST^ (*T*), and the tunneling correction factor, Γ(*T*), which are expressed by Eqs. (4) and (5), respectively. In Eq. (4), Δ*E* is the SCF energy difference between the TS and the local minimum.

3 $$k^{{\rm{SC}}-{\rm{TST}}}\left( T \right) = k^{{\rm{TST}}}\left( T \right) \cdot {\mathrm{\Gamma }}\left( T \right),$$

4 $${k^{{\rm{TST}}}\left( T \right) = \frac{{\mathop {\prod }\nolimits_{i = 1}^N \nu _if(h\nu _i/2k_{\rm{B}}T)}}{{\mathop {\prod }\nolimits_{j = 1}^{N - 1} \nu _j^\dagger f(h\nu _j^\dagger /2k_{\rm{B}}T)}}\; \cdot \;{\mathrm{exp}}\left( { - \frac{{{\mathrm{\Delta }}E}}{{k_{\rm{B}}T}}} \right),f\left( x \right) = \frac{{\sinh \left( x \right)}}{x}},$$

5 $${{\mathrm{\Gamma }}\left( T \right) = \frac{{{\mathrm{exp}}({\mathrm{\Delta }}E_{{\rm{ZP}}}/k_{\rm{B}}T)}}{{1 + {\mathrm{exp}}(2\pi {\mathrm{\Delta }}E_{{\rm{ZP}}}/hv_{{\rm{Im}}}^\dagger )}} + \frac{1}{2}\mathop {\smallint }\nolimits_{ \!\!\!- \infty }^{\pi {\mathrm{\Delta }}E_{{\rm{ZP}}}/h\nu _{{\rm{Im}}}^\dagger } {\rm{d}}\theta {\mathrm{exp}}\left( {\frac{{h\nu _{{\rm{Im}}}^\dagger \theta }}{{\pi k_{\rm{B}}T}}} \right){\rm{sech}}^2\left( \theta \right)}.$$

**Fig. 6:**
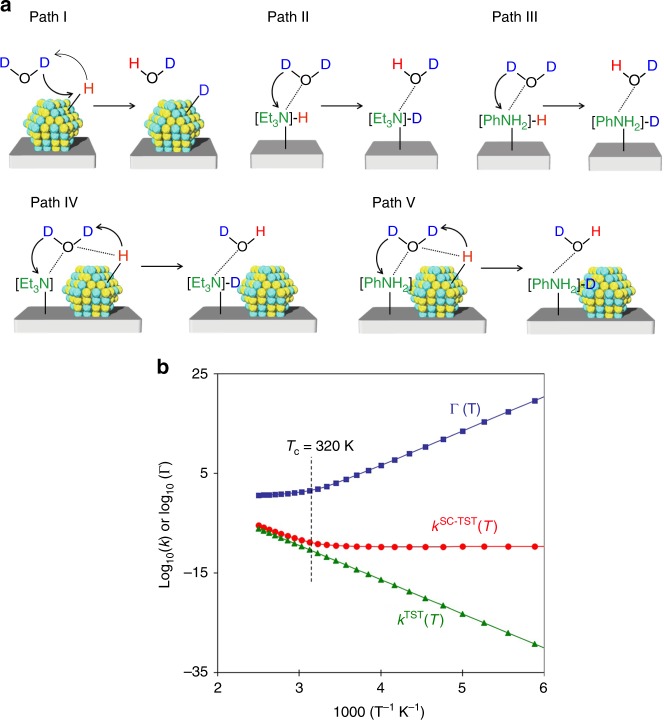
Tunneling effect calculations. **a** Reaction paths used to assess the tunneling effect. (I) direct H/D exchange over the PdAg surface, (II) direct H/D exchange on an NEt_3_ group, (III) direct H/D exchange over a PhNH_2_ group, (IV) H/D exchange over PdAg in the vicinity of an NEt_3_ group and V) H/D exchange over PdAg in the vicinity of a PhNH_2_ group. **b** Arrhenius plots as calculated for path I

The production of hydrogen isotope compounds from an FA/D_2_O combination using PdAg NPs together with amine groups evidently comprises a series of H–D exchange reactions between D_2_O and the PdAg NPs and/or amine groups. Here, five representative reaction paths were considered for the calculations according to the above-proposed reaction mechanism. These include (I) direct H/D exchange over a PdAg surface, (II) direct H/D exchange over an NEt_3_ group, (III) direct H/D exchange over a PhNH_2_ group, (IV) H/D exchange over PdAg in the vicinity of an NEt_3_ group, and (V) H/D exchange over PdAg in the vicinity of a PhNH_2_ group (Fig. 6a).

Table 2 summarizes the *E*_ZP_,*ν*^†^_Im_, and *T*_c_ values determined via Eq. (1) using a Pd_2_Ag_2_ cluster model. Below these values, quantum tunneling dominates the reaction rates, while above these values classical transitions dominate. The *T*_c_ value for path I was determined to be 320 K, which is substantially higher than the values for the other reaction pathways, which range from 123 to 206 K. These values are comparable to those reported for proton-hopping reactions on zeolites (184–320 K)^52^, but slightly lower than that determined for heteropolyacids (353 K)^53^. The *k*^TST^ (*T*) and Γ(*T*) values at various temperatures are also provided in Table 2, while Fig. 6b presents the Arrhenius plots of *k*^TST^ (*T*), Γ(*T*), and *k*^SC-TST^ (*T*) values calculated for path I. Above *T*_c_, *k*^SC-TST^ (*T*), and *k*^TST^ (*T*) are consistent with one another and are also dependent on temperature, meaning that classical transitions dominate. In contrast, *k*^SC-TST^ (*T*) is almost temperature independent below *T*_c_ because of the increase in the tunneling correction factor with decreasing temperature. Owing to the quantum tunneling effect, an Arrhenius fit of the rate data affords different activation energies depending on the temperature range investigated. An activation energy of 46.2 kJ mol^−1^ was obtained from the *k*^SC-TST^ (*T*) data between 300 to 400 K, whereas the values of *k*^SC-TST^ (*T*) from 100 to 200 K gave an activation energy of 0.8 kJ mol^−1^. It should be noted that *k*^SC-TST^ is also larger than *k*^TST^ at *T*_c_ by a factor of more than 35 (Γ(320) = 35.1). Therefore, *T*_c_ is not a distinct point at which a change in mechanism between quantum tunneling and classical transitions occurs, but rather a point where the relative dominance of the two mechanisms rapidly changes^53,63^. For reaction pathways II–V, the temperature independence of *k*^SC-TST^ (*T*) suggests that the tunneling mechanism is predominant (Supplementary Fig. 18), although (as noted) the *T*_c_ values are lower than those for path I, and are below room temperature. Similar trends are observed in the case of the Pd_7_Ag_6_ cluster model, as summarized in Supplementary Table 3 and Supplementary Fig. 19. Here, the *T*_c_ for path I was determined to be 257 K, which is higher than those for paths IV and V. These theoretical investigations strongly suggest that a tunneling mechanism occurs primarily in path I (i.e., direct H/D exchange over the PdAg surface) and plays a crucial role in the overall reaction at temperatures as high as 320 K. The tunneling probability depends to different degrees on the mass of the moving particle, the barrier height, and the barrier width^42^. Especially, the barrier width mainly determines the probability of tunneling in chemical reactions: the narrower a barrier is, the more likely tunneling is^41^. As shown in Supplementary Fig. 20, our calculation results reveal that the *T*_c_ correlates well with *ν*_Im_, not Δ*E*_ZP_. The absolute value *ν*_Im_ is the curvature at the vicinity of the transition state in the reaction coordinate direction, which significantly influence on the barrier width: the larger a value *ν*_Im_ is, the narrower a barrier is. The representative reaction paths used to assess the tunneling effect proceed at the same time; thus, the *T*_c_ of the reaction with highest *T*_c_ can be experimentally observed. The lower *T*_c_ in paths II–V suggests that the H–D exchange at the basic amine cites are largely dominated by classical transitions; thus, the effect of basicity of the amine in the HD/D_2_ ratio can be experimentally appeared.

**Table 2:** Parameters for the calculation of tunneling effect

	Path I	Path II	Path III	Path IV	Path V
Δ*E*_ZP_ (kJ mol^−^^1^)	132.9	215.6	143.3	72.7	86.3
*ν*_Im_ (cm^−1^)	1376.4	731.7	535.3	490	886.7
*T*_c_ (K)	320	168	123	113	206

## Discussion

We succeeded in the controlled synthesis of hydrogen isotope compounds during the dehydrogenation of FA in D_2_O by tuning the H–D exchange reaction, using specific amine-functionalized PdAg nanocatalysts. The grafted amine groups were found to be indispensable to this process, and the reaction selectivity showed a close correlation with the basicity of the amine groups, with the D_2_ selectivity increasing with decreasing basicity. This study provides not only advanced insights into the preferred catalyst architecture but also demonstrates an ideal heterogeneous catalyst system with potential applications to this type of transformation. Large KIE values indicative of the quantum tunneling effect was observed for the reaction of FA in H_2_O vs. D_2_O. Theoretical investigations also emphasized the involvement of a quantum tunneling mechanism, predominantly during the direct H–D exchange reaction over the PdAg surface.

## Methods

### Materials

Pd(NH_3_)_4_Cl_2_ was obtained from the Sigma-Aldrich Chemical Co. AgNO_3_, FA, and 3-aminopropyltriethoxysilane were purchased from Nacalai Tesque, Inc., and aminophenyltrimethoxysilane was obtained from Manchester Organic. All commercially available chemicals were used as received.

### Synthesis of the SA-x series

The mesoporous silica support SBA-15 was prepared using a surfactant self-assembly approach as follows: 4.0 g of Pluronic P123 was dissolved in 30 g of deionized water and 120 mL of 2 M HCl solution with stirring at 313 K; 8.5 g of TEOS was added into the above solution with stirring for 24 h. The mixture was aged at 353 K overnight without stirring, and then the product was recovered by vacuum filtration and washed with deionized water. The resulting white powder was dried at 383 K overnight and calcined at 823 K for 10 h. Physisorbed water was removed before surface modification by drying the SBA-15 at 403 K for 3 h under vacuum. Following this, 100 mL of a toluene solution containing 1.0 g of SBA-15 and the appropriate amount of amine-functionalized silane-coupling reagents was stirred at 373 K for 18 h. The product was recovered by vacuum filtration, washed with toluene and diethyl ether, and then dried under vacuum overnight. An SA-4 sample with Schiff base functional groups was synthesized by the further modification of SA-2 with formaldehyde.

### Synthesis of PdAg/SA-*x*

A quantity of SA-x (0.3 g) was mixed with 50 mL of an aqueous solution containing Pd(NH_3_)_4_Cl_2_ (10 mM, 2.85 mL) and AgNO_3_ (10 mM, 2.85 mL), followed by stirring at room temperature for 1 h. The suspension was evaporated under vacuum and the resulting powder was dried overnight. Subsequently, each sample was reduced with NaBH_4_, giving PdAg/SA-*x* (1 wt% Pd; Pd:Ag molar ratio = 1:1). Analysis using inductively coupled plasma (see Characterization section for details) clearly indicated that the desired amount of each metal species was loaded onto each support.

### Characterization

Powder XRD patterns were recorded using a Rigaku Ultima IV diffractometer with Cu Kα radiation (*λ* = 1.54056 Å). Nitrogen adsorption–desorption isotherms were acquired at 77 K using a BELSORP-max system (MicrotracBEL Corp.). Prior to analysis, samples were degassed at 423 K for 3 h under vacuum to remove physisorbed water. Specific surface areas were calculated using the BET method based on the nitrogen adsorption data. Inductively coupled plasma optical emission spectrometry data were obtained using a Nippon Jarrell-Ash ICAP-575 Mark II instrument. TG analysis was performed by a Rigaku Thermo plus EVO2 TG8121 system from room temperature to 973 K at a heating rate of 10 K min^−1^ in air flow (10 mL min^−1^). TEM micrographs were obtained with a Hitachi Hf-2000 FE-TEM equipped with a Kevex energy-dispersive X-ray (EDX) detector operated at 200 kV. STEM images and elemental maps were obtained with a JEOL-ARM 200F equipped with a Kevex EDX detector (JED-2300T) operated at 200 kV. FT-IR spectra were obtained with a JASCO FT/IR-6100. Samples were prepared for analysis by mixing with KBr, followed by compression into thin disk-shaped pellets. XPS was performed with a Shimadzu ESCA-3400 system, using Mg Kα X-ray radiation (*hν* *=* 1253.6 eV) as the excitation source. The binding energy values for each spectrum were calibrated relative to the contaminant C 1*s* core level binding energy of 284.5 eV. Pd and Ag K-edge XAFS spectra were acquired using a fluorescence-yield collection technique at the 01B1 beamline at SPring-8, JASRI, Harima, Japan, in conjunction with a Si (111) monochromator (proposal numbers 2018A1144 and 2018B1082). Data reduction was performed using the REX2000 program (Rigaku).

### Dehydrogenation of FA

A 0.1 g quantity of catalyst was transferred into a 30 mL reaction vessel containing 1.8 mL D_2_O, after which the vessel was sealed with a rubber septum. The reaction mixture was bubbled with Ar for 15 min, followed by the addition of 0.2 mL of a 5 M solution of HCOOH:HCOONa (9:1) in D_2_O, with magnetic stirring at 343 K. The amounts of H_2_, HD, and D_2_ generated were determined by GC using a Shimadzu GC–2014 instrument with He as the carrier gas and a Shinwa OGO-SP column held at 77 K, in conjunction with a thermal conductivity detector^66^. TOFs in units of h^−1^ were calculated as the total moles of all hydrogen isotopes divided by total moles Pd in the catalyst after 30 min.

### In situ deuteration in the FA-D_2_O system

A 0.1 g quantity of the PdAg/SA-5 was placed into a 30 mL reaction vessel containing 1.8 mL D_2_O and 0.1 mmol of the substrate, after which the vessel was sealed with a rubber septum. A 0.2 mL quantity of a 5 M HCOOH:HCOONa (9:1) solution in D_2_O was added and the reaction mixture was magnetically stirred at 353 K. After 24 h, the reaction mixture was passed through a membrane filter to remove the catalyst. The filtrate was mixed with Et_2_O (10 mL) and the aqueous layer was extracted with Et_2_O (2 × 10 mL), dried over MgSO_4_, filtered, and concentrated in vacuo to give the analytically pure deuterated produces. The yield was determined by GC/mass spectrometry (MS) equipped with capillary column and the D contents of the products were determined by ^1^H nuclear magnetic resonance (NMR) spectroscopy on the basis of the integration of the aromatic protons.

### Computational method

FA adsorption energy values, *E*_ad_, were calculated using DFT, employing the DMol3 program in the Materials Studio 17.2 software package^67^. The generalized gradient approximation exchange-correlation functional proposed by Perdew, Burke, and Ernzerhof was combined with the double numerical basis set plus polarization functions. A supercell slab consisting of a 4 × 4 surface unit cell with three atomic (111) surface layers was adopted, with the bottom two layers fixed at the corresponding bulk positions and the top layers allowed to relax during geometry optimizations. The slab was separated by a vacuum space with a height of 30 Å. *E*_ad_ was defined by the equation *E*_ad_ = *E*_FA_ + *E*_slab_ – *E*_FA/slab_, where *E*_FA_ is the total energy of free FA, *E*_slab_ is the total energy of the bare slab model, and *E*_FA/slab_ is the total energy of the FA–slab system.

DFT calculations to obtain total energy, harmonic frequency, and Gibbs free energy values for the reactants, TSs, and products for the five reactions were performed using the Gaussian 09 program. In each case, TSs were first characterized, after which an intrinsic reaction coordinate analysis was performed to seamlessly connect the TSs to the reactants and products^68^. The Becke three-parameter Lee−Yang−Parr hybrid-type functional^69^ was used, which was implemented in the Gaussian 09 program, and the Los Alamos ECP^70^ with the double-ζ valence basis^71^ was employed for the basis sets of Pd and Ag atoms, while the 6–311 G(d,p) basis sets were used for H, C, N, and O atoms. The *T* values and reaction rates were calculated using both Pd_2_Ag_2_ and Pd_7_Ag_6_ cluster models.

## Supplementary information

Supplementary Information

Peer Review

## Data Availability

All data generated and analyzed during this study are included in this article and its Supplementary Information or are available from the corresponding authors upon reasonable request.
