# Supplementary material for: Controlled release of hydrogen isotope compounds and tunneling effect in the heterogeneously-catalyzed formic acid dehydrogenation
Source: Nat Commun. 2019 Sep 25;10:4094. doi: 10.1038/s41467-019-12018-7 (PMC6761165; doi:10.1038/s41467-019-12018-7)
Supplement: Supplementary file 1 — Supplementary Information [file 41467_2019_12018_MOESM1_ESM.pdf]

Supplementary information

Controlled release of hydrogen isotope compounds and tunneling effect in the heterogeneously-catalyzed formic acid dehydrogenation

Mori et al.

Supplementary Table 1. Textural properties of the PdAg/S and PdAg/SA-x (x=1-5) and adsorption energy (E_{ad}) of formic acid determined by DFT calculation.

Sample	grafted amine / $mmol \cdot g^{-1}$	S_{BET} / $m^2 \cdot g^{-1}$	D_{pore} / nm	V_{pore} / $cm^3 \cdot g^{-1}$	d_{ave} / nm	E_{ad} of FA / $kJ \cdot mol^{-1}$
PdAg/S	-	733	8.9	1.35	7.3	-
PdAg/SA-1	0.3	406	8.9	0.87	7.3	215
PdAg/SA-2	0.3	522	8.9	1.05	4.5	180
PdAg/SA-3	0.3	517	8.3	1.04	6.5	178
PdAg/SA-4	0.3	500	8.0	1.00	4.7	155
PdAg/SA-5	0.3	635	8.3	1.22	4.5	120

Supplementary Table 2. Parameters for the calculation of tunneling effect. Transition state theory (TST) rate constant (k^{TST} (T)) and the tunneling correction factor (Γ(T)) for path I-V using Pd_2Ag_2 model.

	Path I		Path II		Path III		Path IV		Path V	
T / K	$k^{TST}(T)$	$\Gamma(T)$	$k^{TST}(T)$	$\Gamma(T)$	$k^{TST}(T)$	$\Gamma(T)$	$k^{TST}(T)$	$\Gamma(T)$	$k^{TST}(T)$	$\Gamma(T)$
100	1.80×10^{-58}	1.83×10^{48}	2.90×10^{-100}	2.14×10^{46}	3.72×10^{-64}	6.46×10^{14}	9.79×10^{-27}	1.72×10^{5}	2.45×10^{-33}	4.88×10^{23}
200	6.67×10^{-24}	3.12×10^{13}	9.0×10^{-44}	5.57	1.31×10^{-26}	2.06	1.30×10^{-7}	1.80	1.24×10^{-10}	7.86×10
300	1.82×10^{-12}	2.60×10^{2}	6.0×10^{-25}	1.79	4.39×10^{-14}	1.34	3.23×10^{-1}	1.27	4.61×10^{-3}	2.51
400	8.81×10^{-7}	4.08	-	-	-	-	-	-	-	-

Supplementary Table 3. Parameters for the calculation of tunneling effect. Activation barrier (ΔE_{zp}), frequency (ν_{Im}) and tunneling crossover temperature (T_c) for the calculation of tunneling effect and transition state theory (TST) rate constant ($k^{TST}(T)$) and the tunneling correction factor (Γ(T)) for path I, IV and V using Pd_7Ag_6 model.

	Path I	Path IV	Path V
ΔE_{zp}/kJ·mol^{-1}	146.8	82.1	113.7
ν_{Im}/cm^{-1}	1113.0	811	582.1
T_c / K	257	188	134

T / K	$k^{TST}(T)$	$\Gamma(T)$	$k^{TST}(T)$	$\Gamma(T)$	$k^{TST}(T)$	$\Gamma(T)$
100	1.07×10^{-65}	2.14×10^{47}	1.64×10^{-33}	4.98×10^{20}	2.86×10^{-47}	4.87×10^{15}
200	1.42×10^{-27}	1.21×10^{9}	3.50×10^{-12}	1.34×10	2.73×10^{-17}	2.43
300	6.15×10^{-15}	6.08	4.30×10^{-5}	2.10	2.80×10^{-7}	1.42

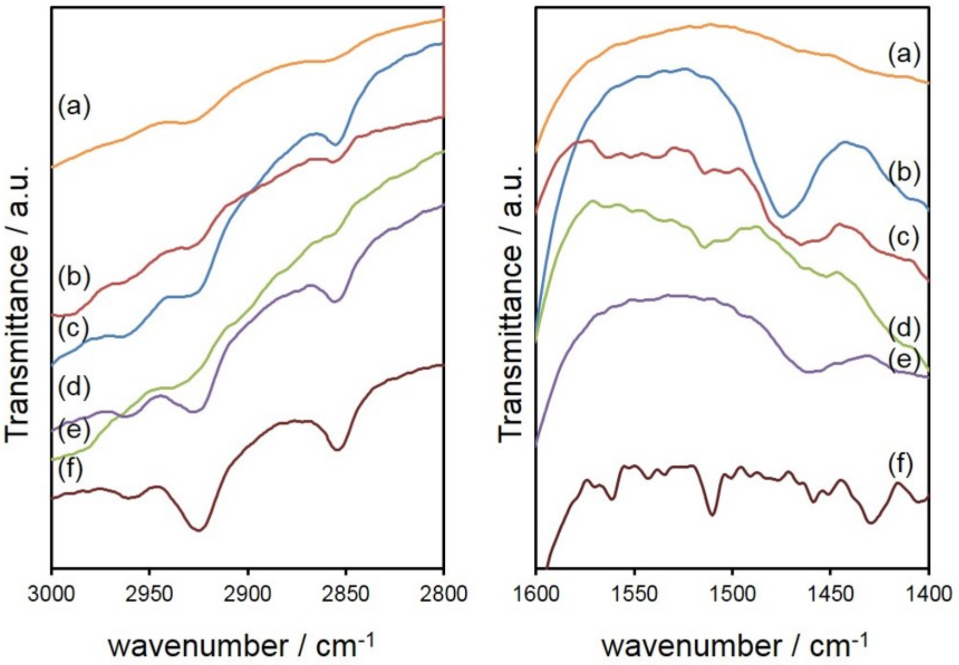

Supplementary Figure 1. FT-IR spectra. (a) PdAg/S and (b-f) PdAg/SA-x (x= 1-5).

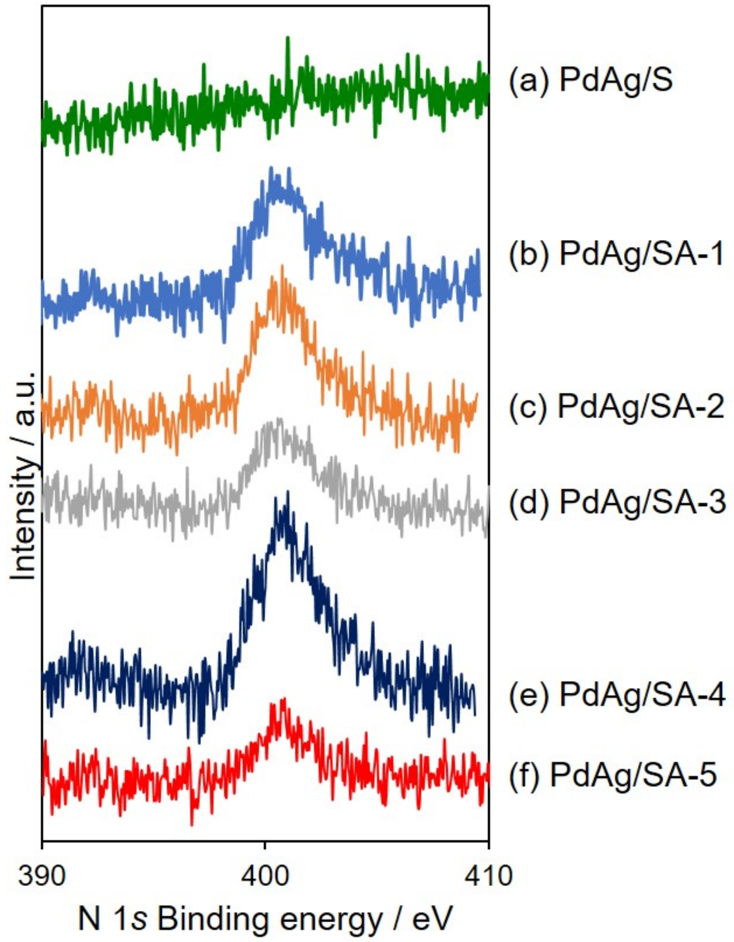

Supplementary Figure 2. N 1*s* spectra. (a) PdAg/S and (b-f) PdAg/SA-x (x= 1-5).

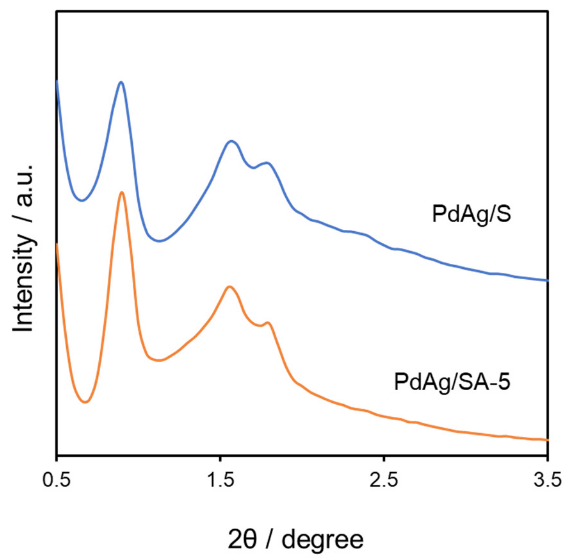

Supplementary Figure 3. Low angle XRD pattern

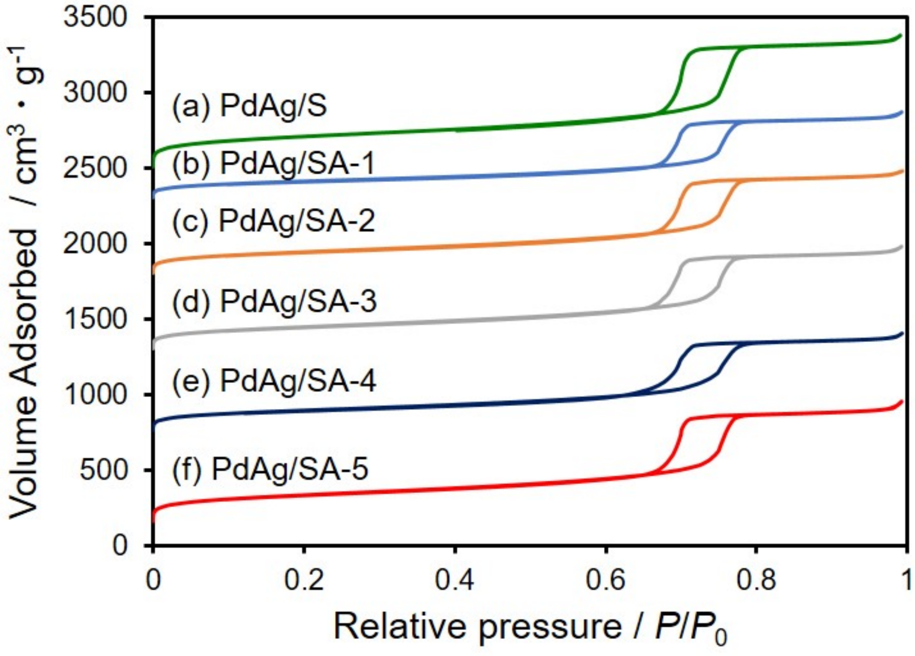

Supplementary Figure 4. N_2 adsorption-desorption isotherm.

(a) PdAg/S and (b-f) PdAg/SA-x (x= 1-5).

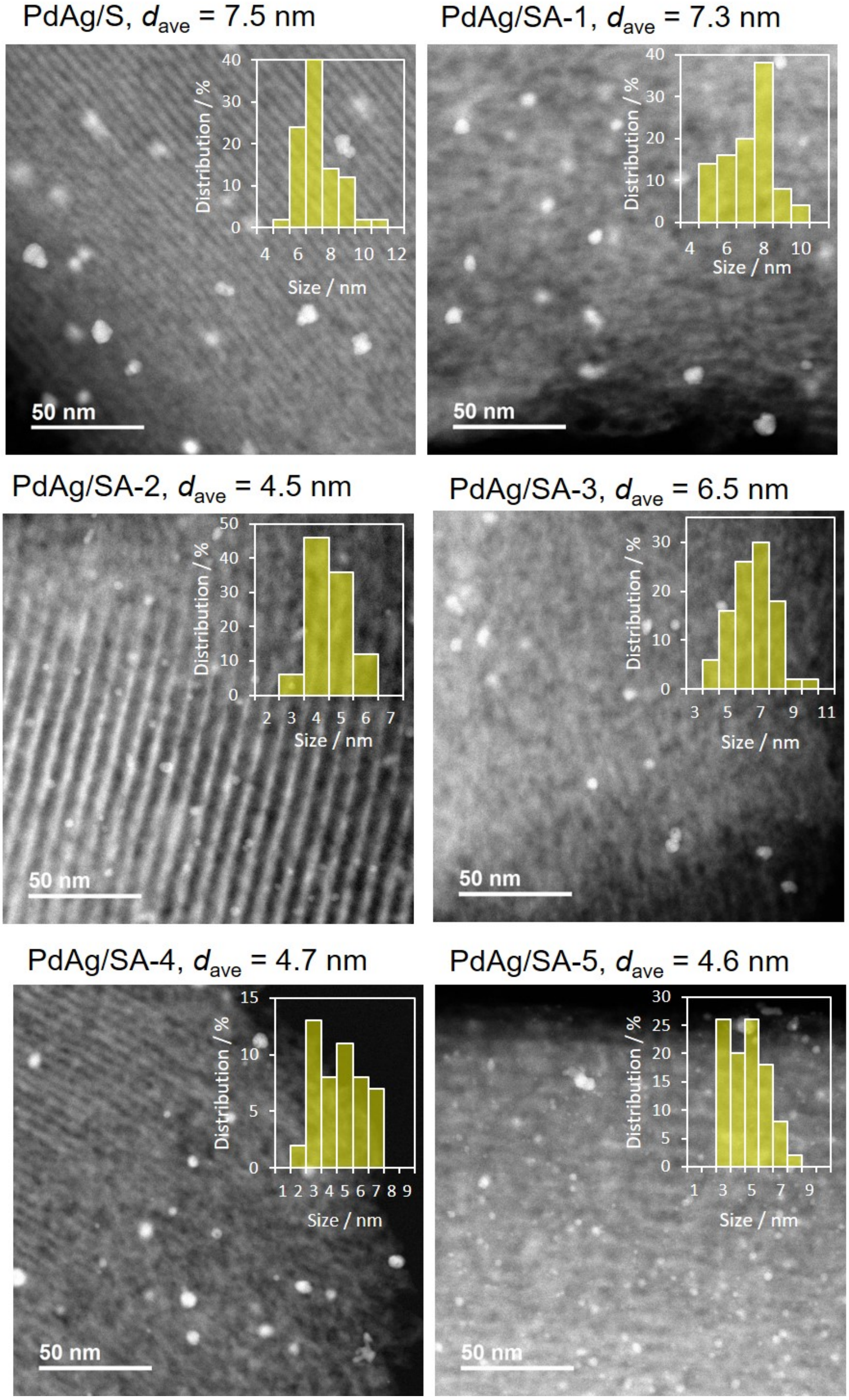

Supplementary Figure 5. HAADF-STEM images and size distribution plots.

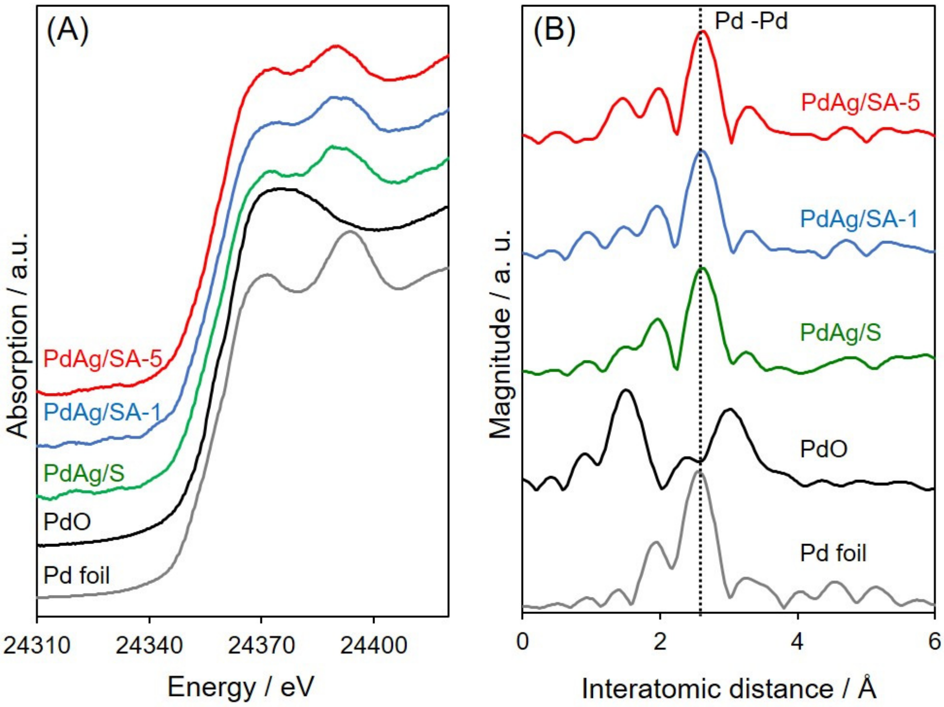

Supplementary Figure 6. Pd K-edge XAFS. (A) XANES spectra and (B) FT-EXAFS spectra.

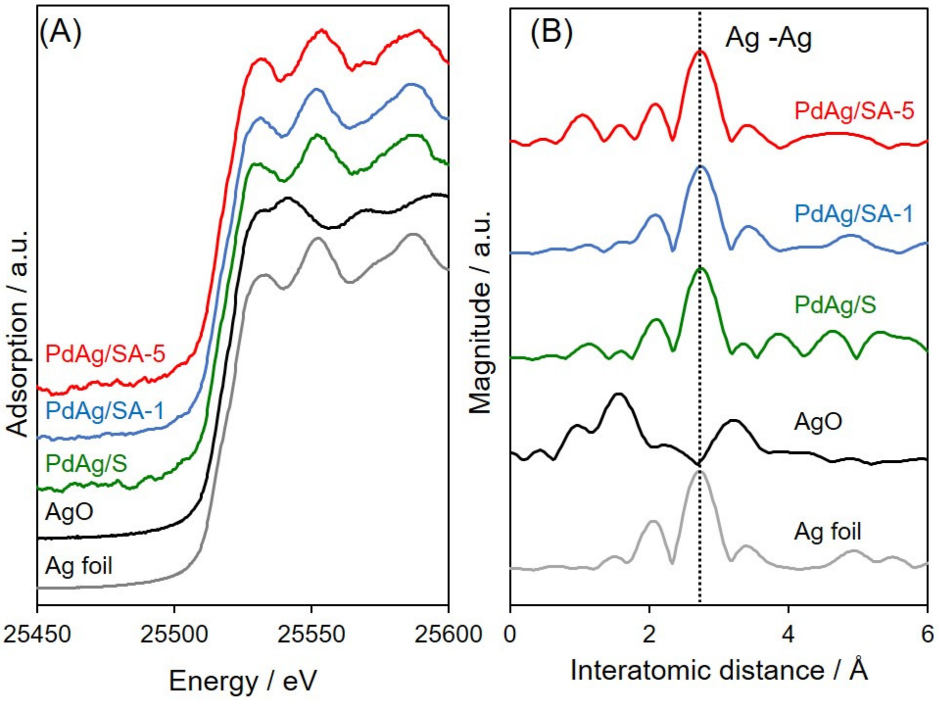

Supplementary Figure 7. Ag K-edge XAFS. (A) XANES spectra and (B) FT-EXAFS spectra.

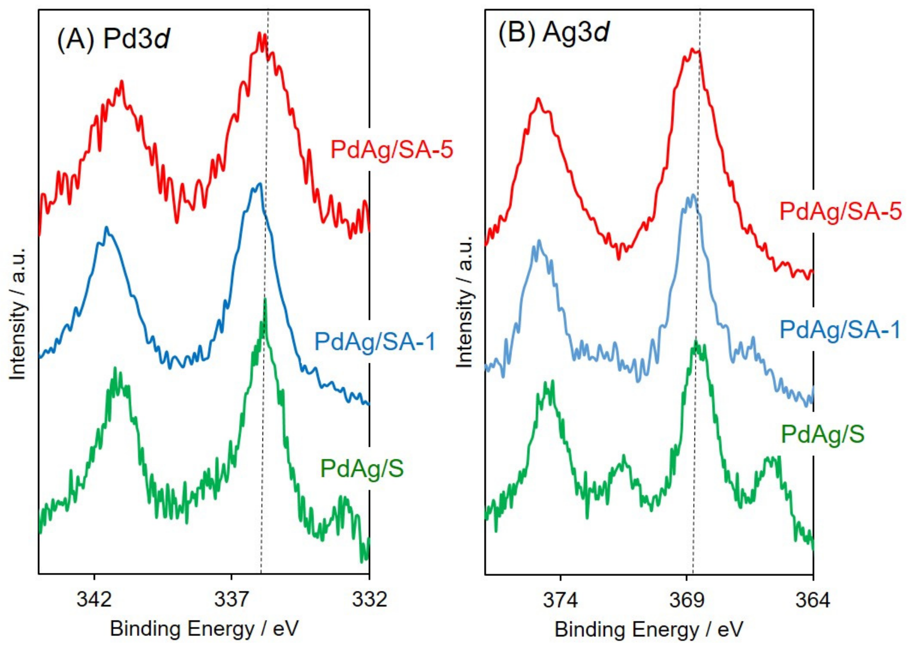

Supplementary Figure 8. XPS spectra. (A) Pd 3*d* and (B) Ag 3*d*.

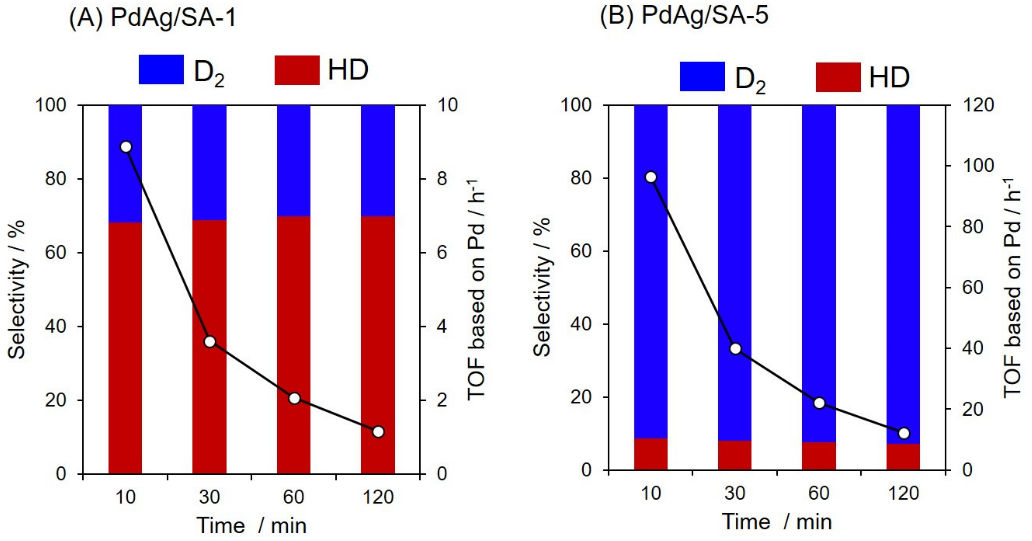

Supplementary Figure 9. Effect of reaction time on the selectivity. (A) PdAg/SA-1 and (B) PdAg/SA-5.

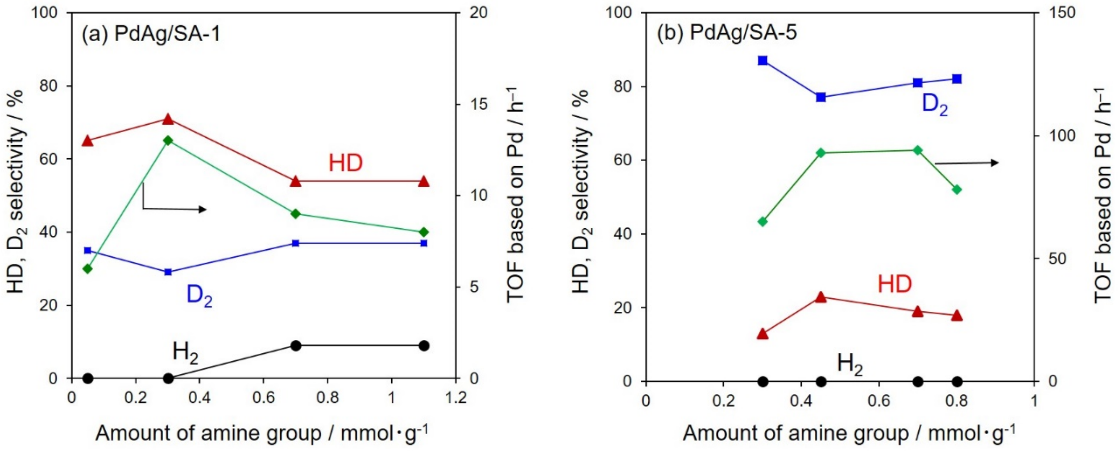

Supplementary Figure 10. Effect of amount of grafted amine groups. (a) PdAg/SA-1 and (b) PdAg/SA-5.

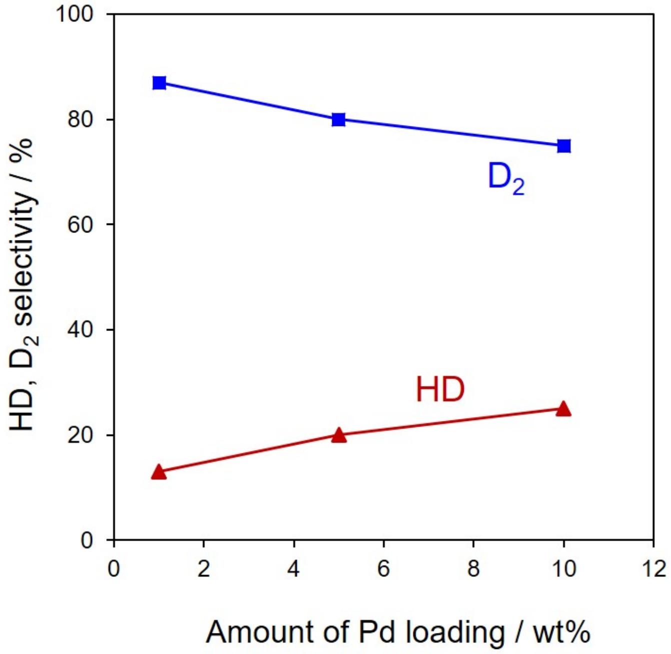

Supplementary Figure 11. Effect of amount of Pd loading for PdAg/SA-5.

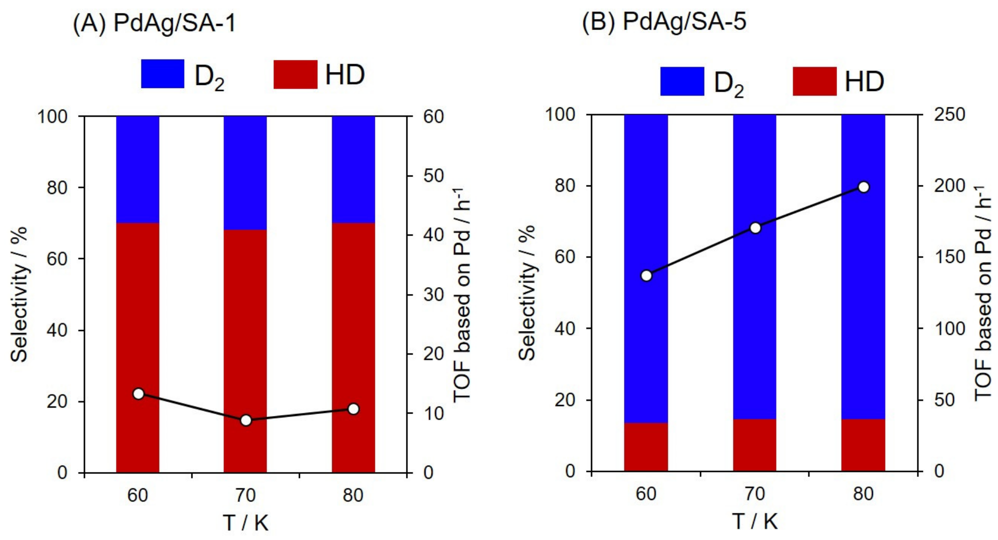

Supplementary Figure S12. Effect of temperature. (A) PdAg/SA-1 and (B) PdAg/SA-5. TOF was determined after 10 min.

]

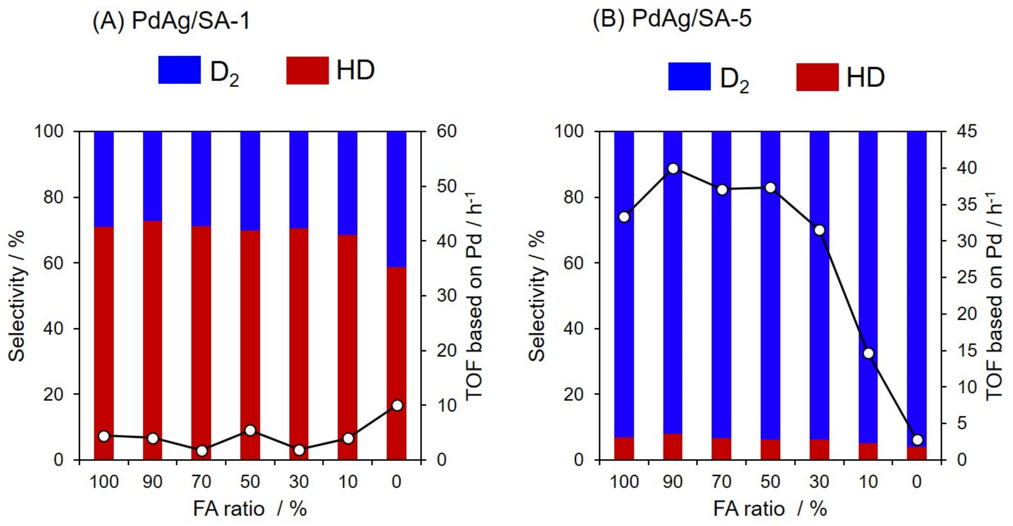

Supplementary Figure 13. Effect of ratio of FA to sodium formate. (A) PdAg/SA-1 and (B) PdAg/SA-5.

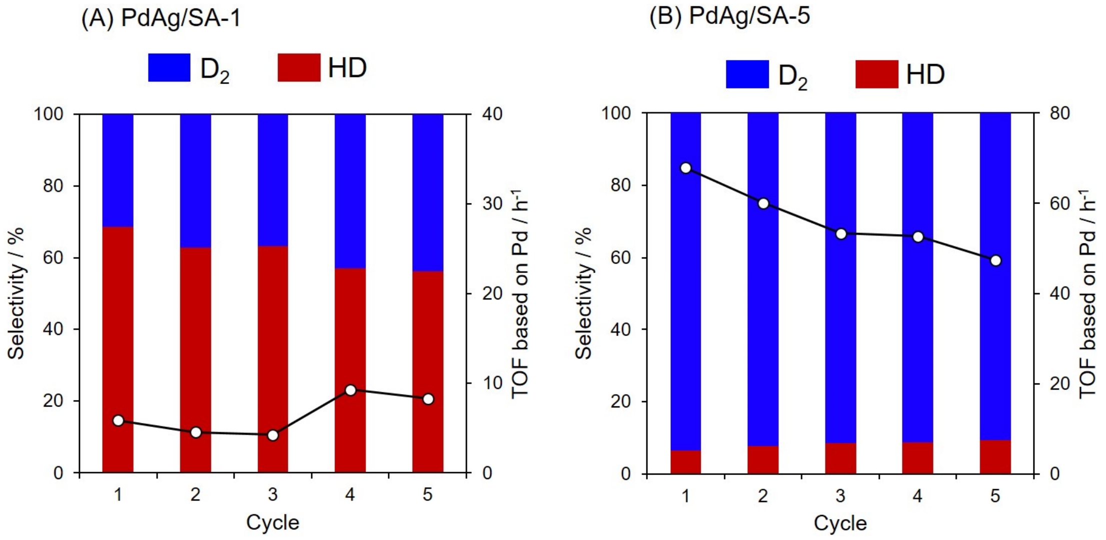

Supplementary Figure S14. Recycling experiments.(A) PdAg/SA-1 and (B) PdAg/SA-5.

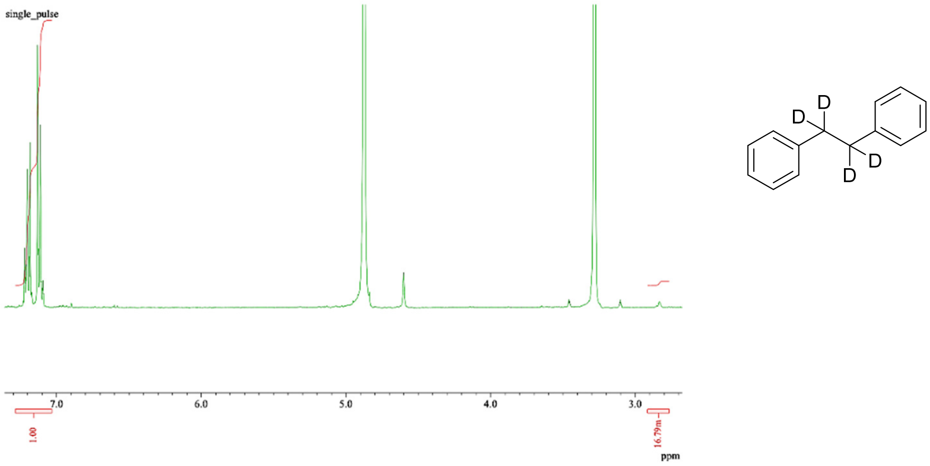

^{1}H NMR (CD_3OD) δ 2.83 (s, 0.02H), 7.09–7.22 (m, 1H); MS (EI) m/z (%): 186 (14.6) $[M(d_4)]^+$, 185 (2.6) $[M(d_3)]^+$, 184 (1.0) $[M(d_2)]^+$, 183 (0.3) $[M(d_1)]^+$, 182 (0.7) $[M(d_0)]^+$, 93 (100), 92 (13).

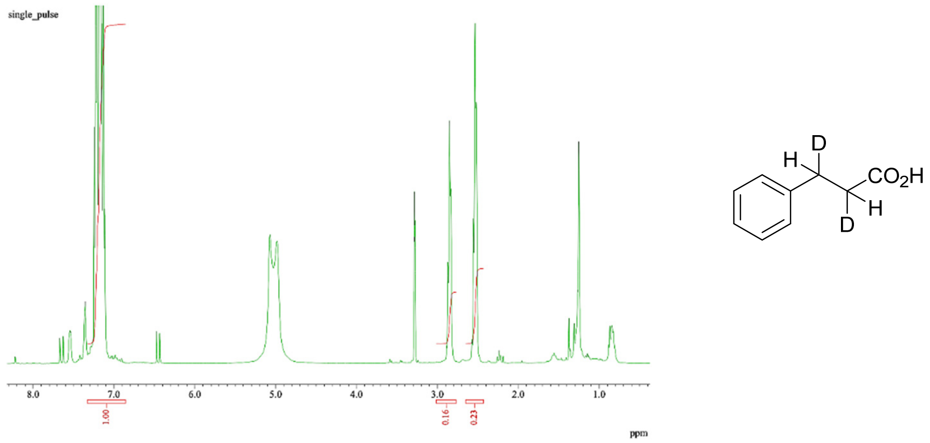

^{1}H NMR (CD_3OD) δ 2.52-2.57 (m, 0.23H), 2.84-2.89 (m, 0.16H), 7.11–7.24 (m, 1H); MS (EI) m/z (%): 152 (22.2) $[M(d_2)]^+$, 151 (6.6) $[M(d_1)]^+$, 150 (0.9) $[M(d_0)]^+$.

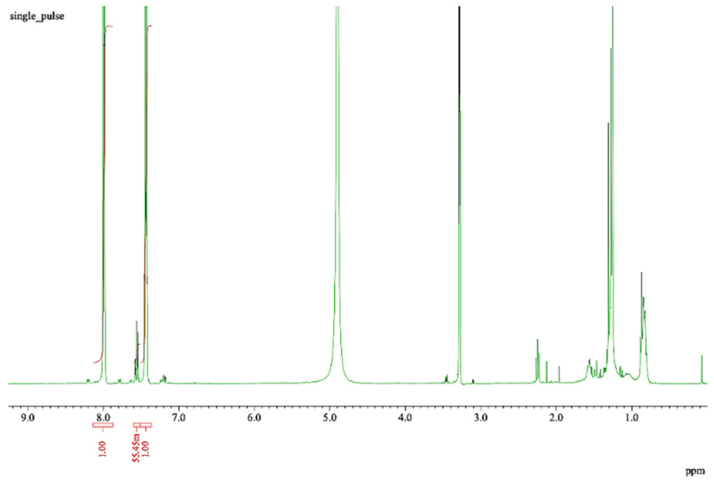

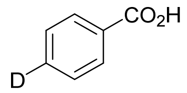

^{1}H NMR (CD_3OD) δ 7.40–7.46 (m, 1H), 7.54-7.58 (m, 0.06H), 7.99–8.00(m, 1H); MS (EI) m/z (%): 123 (52.3) $[M(d_1)]^+$, 122 (6.8) $[M(d_0)]^+$, 106 (100), 105 (15), 78 (91).

Supplementary Figure 15. ^{1}H NMR spectra of the deuterated products. The reactions were performed by using PdAg/SA-5 and 5 M HCOOH:HCOONa (9:1) solution in D_2O.

No.	PVP/Pd [mol/mol]	Temp. [°C]	Pd amount	d_{ave} [nm]
1	10	100	X/4	2.8
2	10	100	X	3.1
3	without PVP, $NaBH_4$ reduction			3.9
4	1	150	X	5.7
5	0.01	100	X	16

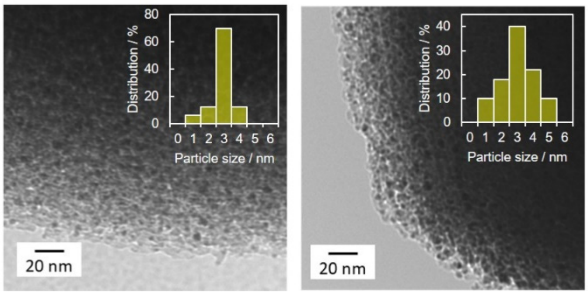

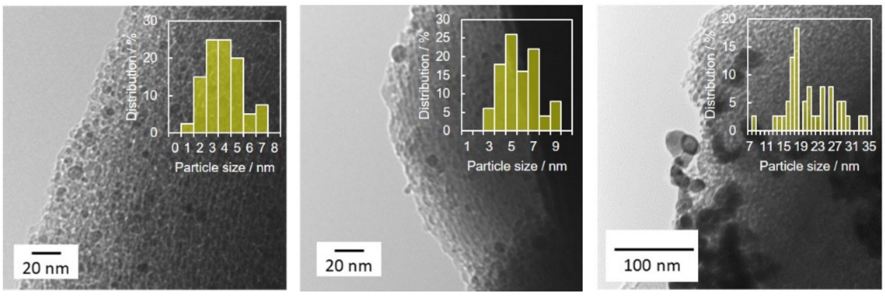

Supplementary Figure 16. Characterization of Pd samples 1-5. TEM images and size distribution diagrams of Pd samples prepared under different synthetic conditions.

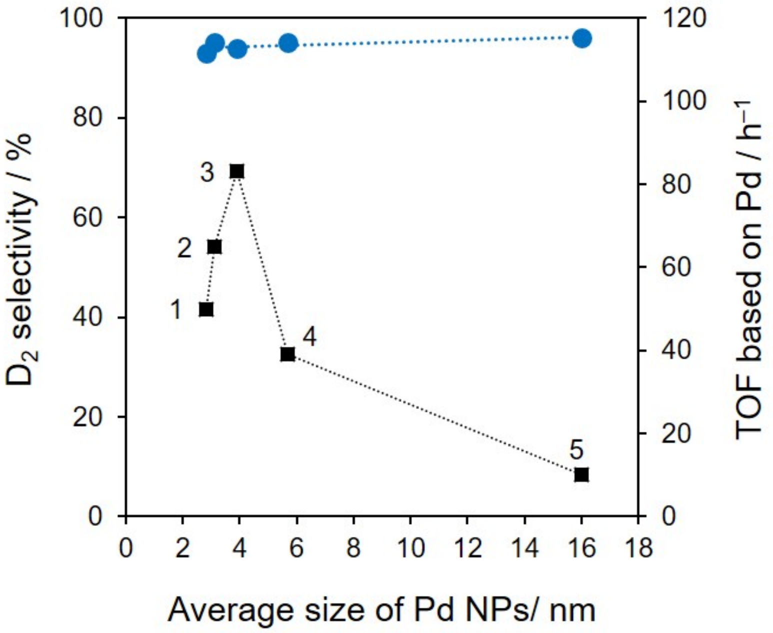

Supplementary Figure 17. Effect of Pd particle size. Average diameters were determined by TEM images for Pd/SA-5 prepared under different synthetic conditions..

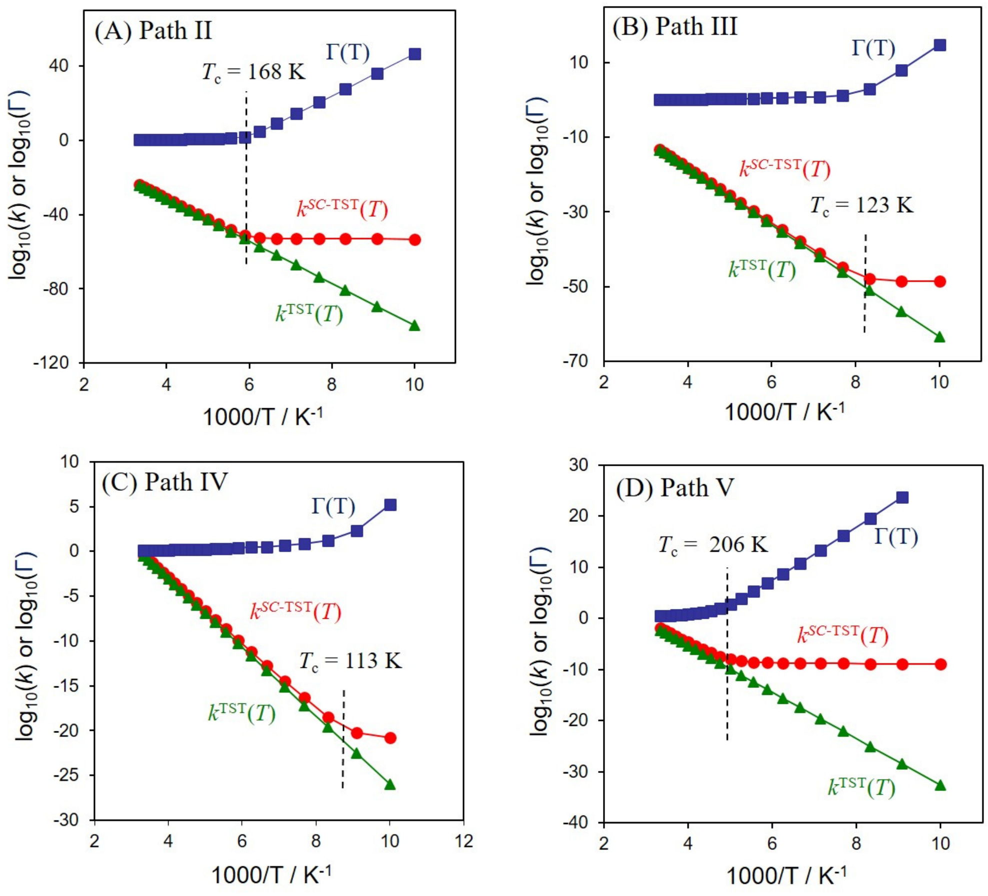

Supplementary Figure 18. Arrhenius plots. $k^{\mathrm{TST}}(T)$, Γ(T), and $k^{\mathrm{SC\text{-}TST}}(T)$ calculated in (A) path II, (B) path III, (C) path IV, and (D) path V using Pd_2Ag_2 model.

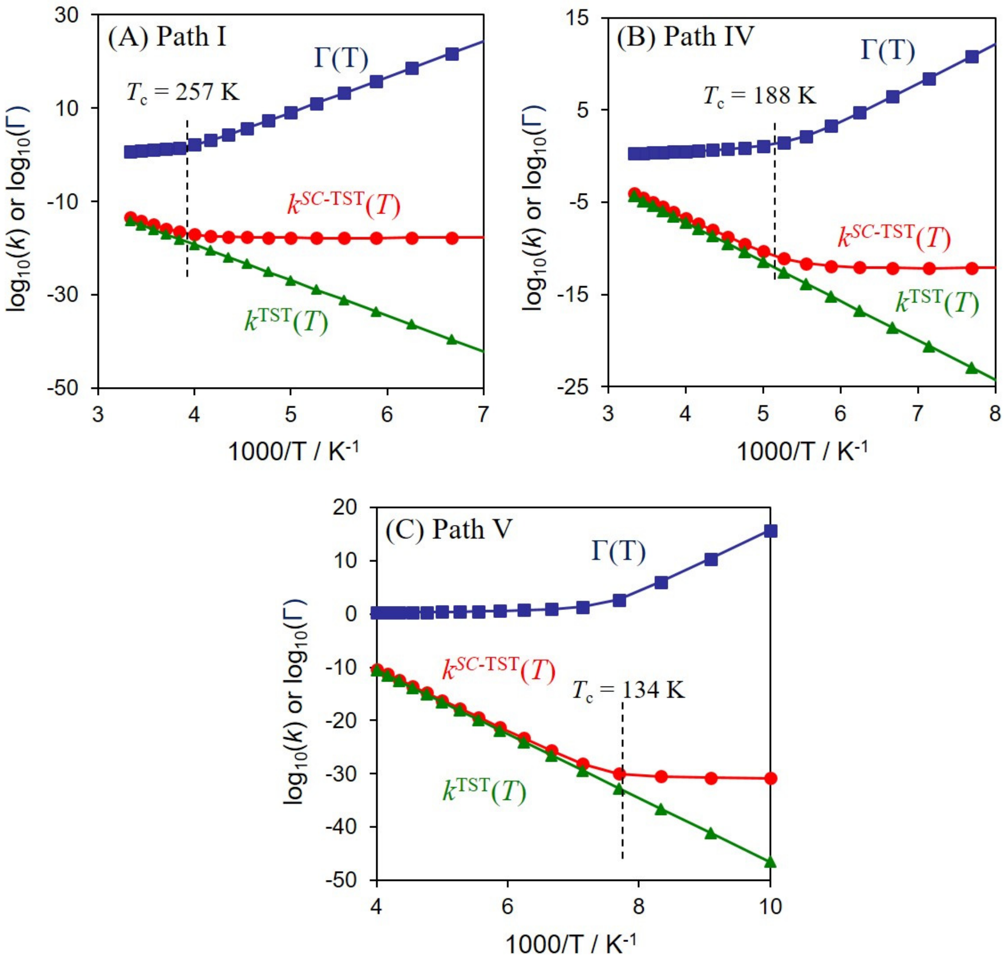

Supplementary Figure 19. Arrhenius plots. $k^{TST}(T)$, Γ(T), and $k^{SC\text{-}TST}(T)$ calculated in (A) path I, (B) path IV, and (C) path V, using Pd_7Ag_6 model.

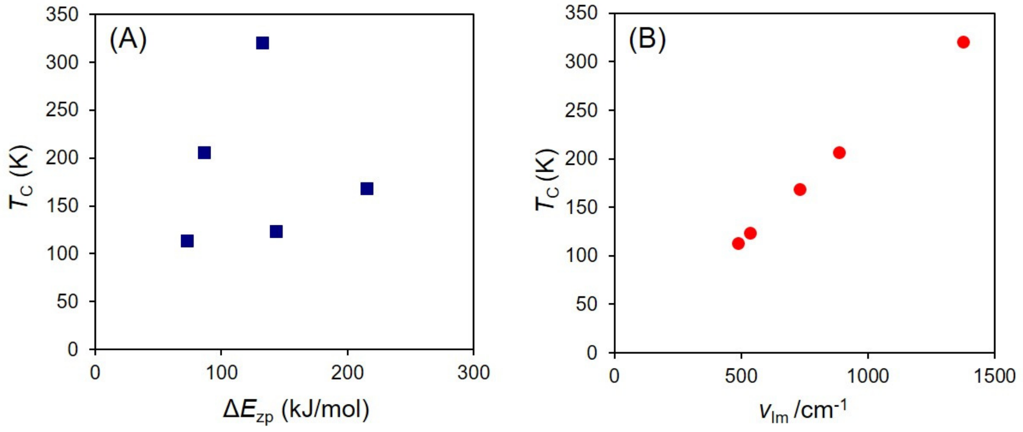

Supplementary Figure 20. Correlation between tunneling crossover temperature (T_c) and (A) activation barrier (ΔE_{zp}) and (B) frequency (v_{Im}).
