# Supplementary material for: Controlled release of hydrogen isotope compounds and tunneling effect in the heterogeneously-catalyzed formic acid dehydrogenation
Source: Nat Commun. 2019 Sep 25;10:4094. doi: 10.1038/s41467-019-12018-7 (PMC6761165; doi:10.1038/s41467-019-12018-7)
Supplement: Supplementary file 2 — Peer Review [file 41467_2019_12018_MOESM2_ESM.pdf]

Reviewers' comments:

Reviewer #1 (Remarks to the Author):

Recommendation:
Major revision

Comments:
This paper reports the production of hydrogen isotope compounds (HD or D2) through formic acid (FA) dehydrogenation reaction in deuterated water (D2O) with PdAg nanocatalysts on a silica substrate whose surface is modified with amine groups. Interestingly, the selectivity of the reaction depended on the basicity of surface amine groups. Weakly basic amine moieties promoted the formation of D2, while strongly basic amine groups switched the selectivity from D2 to HD. This work is the first example to produce hydrogen isotope compounds through heterogeneously catalyzed decomposition of undeuterated FA in relatively cheap D2O solvent. The authors suggested how the selectivity can be controlled by the basicity of surface amines with DFT calculations. They explained that it is relatively kinetically hard to exchange H captured by amines with D of deuterated water with strong amines. The experimental results are interesting. However, following issues should be addressed before the publication of this work.

1) As shown in Figure 5, the authors found that the adsorption energy of FA and HD selectivity is inversely correlated. However, the adsorption energy was completely excluded when the authors explained the control of selectivity (Figure 7).
2) In the proposed mechanism shown in Figure 7, the first H/D exchange occurs between the H on PdAg and the D of solvent. Did the authors consider the possibility that the first H/D exchange occurs between the H at base and the D of solvent?
3) The influence of reaction temperature, reaction time, and the ratio of FA to sodium formate on the selectivity should be discussed.
4) The recyclability of the catalysts should be checked.
5) In DFT calculations, a supercell slab model consisting of (111) surface layers was employed. However, this is not realistic for modeling the surface of the prepared PdAg nanoparticles.
6) Some typos, such as TON in line 197, should be corrected.

Reviewer #2 (Remarks to the Author):

NCOMMS-19-17661
Title: Controlled Release of Hydrogen Isotope Compounds and Tunneling Effect in the Heterogeneously-Catalyzed Dehydrogenation of Formic Acid

The work from Yamasita et al. describes the formation of isotope compounds of hydrogen by a process coupled to the decomposition of formic acid (not enriched in deuterium) in D2O. The process is catalytic, using a PdAg / SA-x heterogeneous catalysts of mesoporous nature doped with Pd / Ag and modified on the surface with aminated groups.
In general, the work is clearly written, and the explanations and conclusions are consistent with the experimental results. The catalysts are well characterized, and the description of their preparation provides enough information for its reproducibility by interested readers.
The speed of the conversions reached (TOF) are not spectacular and many previous works show better results in this regard. This opens up the possibility of improving the catalytic systems used in this draft in later work.
Discrete production of hydrogen is obtained in the decomposition of formic acid under soft conditions. The isotope distribution of deuterium in the hydrogen produced variable with HD/D2 ratios depending on the medium and the nature of the catalysts.

The ability to modulate the ratio of the HD/D2 compounds with the basicity of the modifying amine groups is the most important contribution of this work. This aspect can inspire the preparation of heterogeneous or homogeneous catalysts more active in this type of process.
This work deserves to be published in Nature Communications taking into account the novelty that it is involved in the production of hydrogen isotope compounds and in the possible future application of this technique.
Aspects to improve the article are:
- The description of the processes of deuteration of substrates in table 1 and Figure S10 should be improved and the experimental processes of analysis of the deuterium content must be better described in the experimental section.
- The theoretical studies on the mechanism show some obscure points that the authors must clarify or extend:
i) What do the authors mean by the configuration described in the line 213?
ii) The energies calculated in this section for the H/D exchange between the amine and H2O groups seem too high. The exchange of protons between an ammonium salt and water protons must be a very fast process. Can the authors give an explanation to this?
iii) In these calculations the energy of the H2 formation process through the coupling of the hydride of the metallic nanoparticle and the proton centered in the amine has not been estimated. This information would be of interest to know which the limiting step of the process is.
-Since the quantum tunneling mechanism is predominantly manifested in the direct H-D exchange on the surface of metallic nanoparticles, how can the observed experimental effect of the basicity of the amine in the HD/D2 ratio be understood? This apparent discrepancy between theory and experimental observations must be clarified in the text.

Reviewer #3 (Remarks to the Author):

The paper reports the synthesis of PdAg/SA-x ,and study their controlled release of hydrogen isotope compounds and tunneling effect in dehydrogenation of FA.This researsh is of interest to other researshers. However, the dehydrogenation of FA has been studied in many previous works, and I cannot find the original innovation from this paper. Therefore, I cannot recommend this paper to be published in Nature Communications. Some other comments as follows.
1)For XANES spectra and FT-EXAFS spectra in Figure S5 and S6,why chose PdAg/SA-5 and PdAg/SA-1 as the example? Does two of them have any representation?
2)Is this sentence “It should also be noted that surface amine functional groups were present at the peripheries of the loaded PdAg NPs to alter their electronic state” based on experiments or articles ?
3) A writren error of “TON” in line 197 should be corrected to “TOF”.
4) To prove that the selectivity for the production of hydrogen isotope compounds is not governed by the inherent ability of the metal NPs themselves, a bare SA-5 substrate should be tested for the selectivity. Otherwise , the results can only suggest that the selectivity is irrelevant to the interaction between bimetals.
5) In Figure 5, PdAg/S also should be tested for the relation between the basicity and selectivity to prove the point that hat the basicity of the amine functional groups plays a crucial role in determining selectivity.

Answer to the comments by Reviewer #1

Comment 1: As shown in Figure 5, the authors found that the adsorption energy of FA and HD selectivity is inversely correlated. However, the adsorption energy was completely excluded when the authors explained the control of selectivity (Figure 7).

Answer 1: We would like to thank his/her critical comment. In **Figure 5**, the adsorption energy (E_{ad}) of FA was used as a criterion of the basicity of the modified amine functional groups, since the adsorption of FA involves formation of an acid–base pair via N–H···O hydrogen bonding. E_{ad} over SA-1 with strongly basic −$N(C_2H_5)_2$ groups was found to be -215 kJ·mol^{-1}, which was substantially stronger than the value of -120 kJ·mol^{-1} over SA-5, having –weakly basic $PhNH_2$ groups. In **Figure 7**, the activation energy (E_a) for the H-D exchange reaction at the strongly basic −$N(C_2H_5)_2$ site is 212.5 kJ/mol, which is larger than 149.8 kJ/mol for the H-D exchange reaction at the weakly basic −$PhNH_2$ site. These results clearly suggest that the adsorption energy of FA influence on the activation energy for the H-D exchange reaction at the basic site, which finally determine the selectivity of the hydrogen isotope compounds.

In order to clarify the meaning of adsorption energy of formic acid, the caption of X axis in **Figure 5A** was changed to "Basicity of the amine groups determined by E_{ad} of FA".

Furthermore, the following sentence was added to the text, in order to clarify the above discussions.

(Page 8) The E_a for the H-D exchange reaction at the strongly basic −$N(C_2H_5)_2$ site is substantially larger than that at the weakly basic −$PhNH_2$ site, which are reflected by the basicity determined by the E_{ad} of FA in **Figure 5A**; the E_a increased with increasing the E_{ad} of FA. It can therefore be concluded that the selectivity for hydrogen isotope compounds is determined by the basicity of the surface amine groups, which ultimately influence on the H-D exchange reaction at the basic sites in the peripheries of the active centers.

Comment 2: In the proposed mechanism shown in Figure 7, the first H/D exchange occurs between the H on PdAg and the D of solvent. Did the authors consider the possibility that the first H/D exchange occurs between the H at base and the D of solvent?

Answer 2: We would like to thank his/her critical comment. As shown in **Figure 7**, in the case of PdAg/SA-1 with strongly basic −NEt_2 groups, the E_a for the H/D exchange between the H at base and the D of D_2O (from **5** to **6**) is calculated to be 212.5 kJ/mol, which is larger than 140.2 kJ/mol for the H/D exchange between the H at PdAg and the D of D_2O (from **4** to **5**). This suggest that the former reaction hardly occur prior to the later reaction, which explains the preferential formation of

HD. On the other hand, the E_a for both H/D exchange reactions are quite similar in the case of PdAg/SA-5 with weakly basic −$PhNH_2$ groups (140.2 kJ/mol vs. 149.8 kJ/mol). Thus, there is the possibility that the H/D exchange between the H at base and the D of D_2O (from **8** to **9**) occurs prior to that between the H at PdAg and the D of D_2O (from **7** to **8**). Even so, the latter reaction subsequently occurs to afford reaction intermediate **9** because of their similar E_a, which ultimately allows the predominant formation of D_2.

The above discussion was clarified in the revised manuscript as follows.

(Page 8) In the case of PdAg/SA-1, which has strongly basic −NEt_2 groups, **4** preferentially undergoes an H-D exchange reaction at the Pd site with a barrier of E_a = 140.2 kJ/mol to afford **5**. Subsequently, an H-D exchange reaction proceeds at the basic amine site with a barrier of 212.5 kJ/mol to produce **6**. The intermediates **5** and **6** release HD and D_2, respectively. Thus, the substantially higher activation energy for the formation of **6** compared to that for **5** contradict the occurrence of the reversed reaction, which also explains the preferential formation of HD. In contrast, the activation energies for the formations of **8** and **9** from **7** via the H-D exchange reaction at Pd and weakly basic −$PhNH_2$ groups in the case of PdAg/SA-5 were calculated to be 140.2 and 149.8 kJ/mol, respectively. From an energetic point of view, the H-D exchange reaction would therefore be expected to occur simultaneously at both sites, leading to the preferential formation of intermediate **9** and ultimately favoring D_2 generation.

Comment 3: The influence of reaction temperature, reaction time, and the ratio of FA to sodium formate on the selectivity should be discussed.

Answer 3: The influence of some parameters for the reaction conditions, such as reaction temperature, reaction time, and ratio of FA to sodium formate, on the selectivity of the hydrogen isotope compounds were investigated. As shown in **Figure S8, S11, and S12**, the selectivities were almost independent on such parameters, although change of TOFs were observed. In order to clarify these results, the following sentences were added in the text and **Figure S8, S11, and S12** were added in Supporting Information.

(Page 6) The D_2 and HD selectivity appeared to be independent of the yield level and remained unchanged during the reaction, suggesting that the H-D exchange reaction with the produced hydrogen isotope compounds did not take place (**Figure S8**).

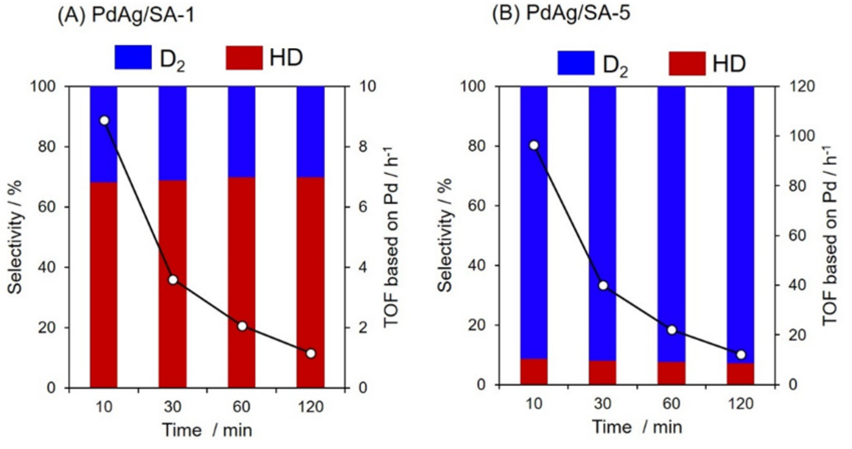

Figure S8. Effect of reaction time on the hydrogen isotope selectivity for (a) PdAg/SA-1 and (b) PdAg/SA-5.

(Page 6) In contrast, the type of amine functional group, PdAg NP loading, reaction temperature, and ratio of FA to sodium formate did not show significant effect on selectivity (**Figures S9-S12**), demonstrating that these parameters are minor factors in determining the selectivity.

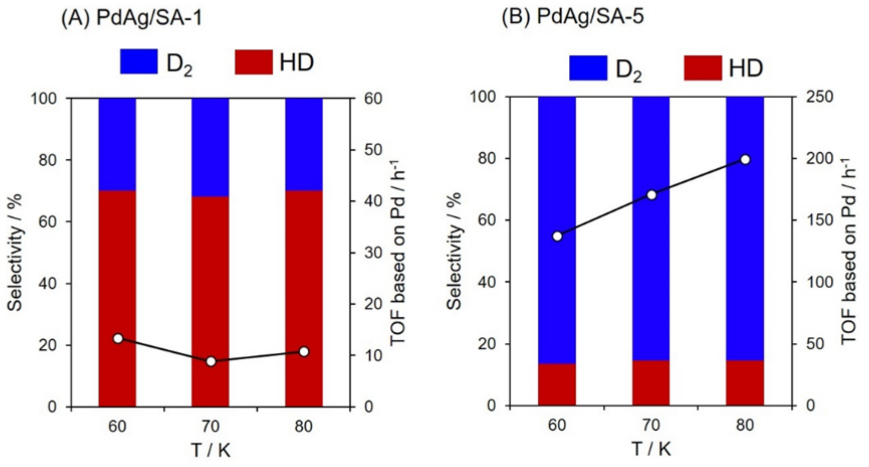

Figure S11. Effect of temperature on the hydrogen isotope selectivity for (a) PdAg/SA-1 and (b) PdAg/SA-5. TOF was determined after 10 min.

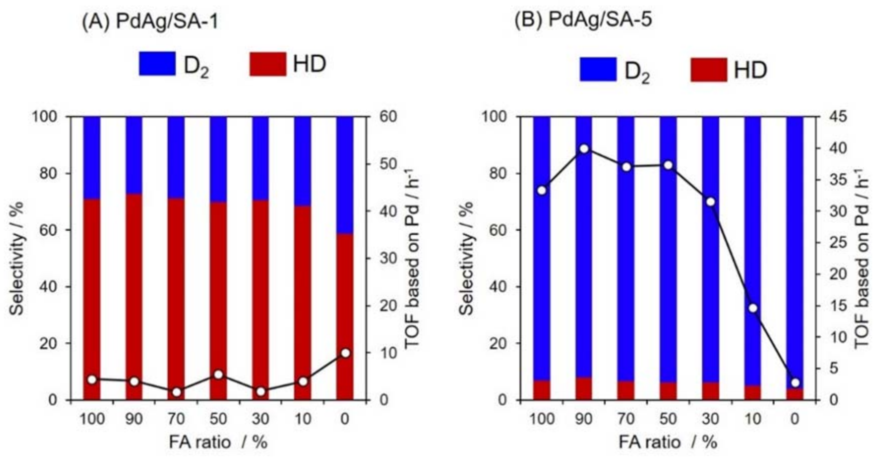

Figure S12. Effect of ratio of FA to sodium formate on the hydrogen isotope selectivity for (a) PdAg/SA-1 and (b) PdAg/SA-5.

Comment 4: The recyclability of the catalysts should be checked.

Answer 4: The recyclability is a crucial point to consider when heterogeneous catalysts are employed for industrial applications. The results are shown in **Figure 13**. After the reaction, the PdAg/SA-x (x= 1 and 5) catalysts were easily separated from the reaction mixture and could be reused with retention of its activity and selectivity; preferential formation of hydrogen isotope compounds could be attained while keeping their initial reaction rates for at least five recycling experiments.

In order to clarify these results, the following sentences were added in the text.

(Page 6) The recyclability is a crucial point to consider when heterogeneous catalysts are employed for industrial applications. After the reaction, the PdAg/SA-x (x= 1 and 5) catalysts were easily separated from the reaction mixture and could be reused with retention of its activity and selectivity; preferential formation of hydrogen isotope compounds could be attained for at least five recycling experiments (**Figure S13**).

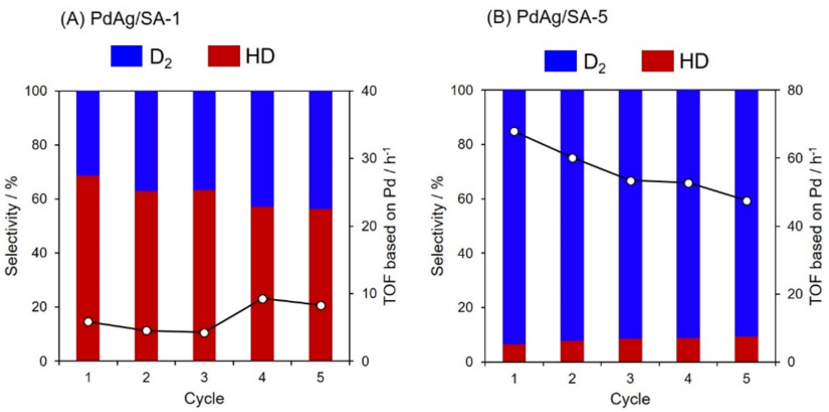

Figure S13. Results of recycling experiments for (a) PdAg/SA-1 and (b) PdAg/SA-5.

Comment 5: In DFT calculations, a supercell slab model consisting of (111) surface layers was employed. However, this is not realistic for modeling the surface of the prepared PdAg nanoparticles.

Answer 5: It is well known that the (111) facet seems to be exposed in clusters of FCC metals and alloys because the surface atomic density is highest and the surface is stable for the (111) surface. The authors have employed the (111) facet clusters in many calculation works of FCC metals.

1. "Density Functional Study of Ethylene Oxidation on an Ag(111) Surface", Hisayoshi Kobayashi, Katsumi Nakashiro and Tomoatsu Iwakura, *Theor. Chem. Acc.*, **102**, 237-243 (1999).
2. "Density Functional Study of Ethylene Hydrogenation on Pt(111) Surface", Toshiko Miura, Hisayoshi Kobayashi and Kazunari Domen, *J. Phys. Chem. B*, **104**, 6809-6814 (2000).
3. "Density Functional Study of Ethylene Oxidation on Ag(111) Surface. Machanism of Ethylene-Oxide Formation and Complete Oxidation with Influence of Subsurface Oxygen", Hisayoshi Kobayashi, Katsumi Nakashiro and Tomoatsu Iwakura, *Inter. Elec. J. Mol. Design*, **1**, 620-635 (2002).
4. "Density Functional Study of Propylene Oxidation on Ag and Au Surfaces. Comparison to Ethylene Oxidation", Hisayoshi Kobayashi and Yoshiki Shimodaira, *J. Mol. Struc.: THEOCHEM*, **762**, 57-67 (2006).
5. "Density Functional Theory Study of Anode Reactions on Pt-Based Alloy Electrodes", Yoshiki Shimodaira, Toshitaka Tanaka, Toshiko Miura, Akihiko Kudo, and Hisayoshi Kobayashi, *J. Phys. Chem. C*, **111**[1], 272-279 (2007).

Comment 6: Some typos, such as TON in line 197, should be corrected.

Answer 6: We would like to thank his/her comment. The "TON" was changed to "TOF". (page 7)

Answer to the comments by Reviewer #2

Comment 1: The description of the processes of deuteration of substrates in Table 1 and Figure S10 should be improved and the experimental processes of analysis of the deuterium content must be better described in the experimental section.

Answer 1: In order to clarify the more detail experimental conditions and processes, the reaction time and the reaction temperature were added in **Table 1**. The experimental section was modified as follows.

(Page 14) ***In situ* deuteration in the FA-D_2O system:** A 0.1 g quantity of the PdAg/SA-5 was placed into a 30 mL reaction vessel containing 1.8 mL D_2O and 0.1 mmol of the substrate, after which the vessel was sealed with a rubber septum. A 0.2 mL quantity of a 5 M HCOOH:HCOONa (9:1) solution in D_2O was added and the reaction mixture was magnetically stirred at 353 K. After 24 h, the reaction mixture was passed through a membrane filter to remove the catalyst. The filtrate was mixed with Et_2O (10 ml) and the aqueous layer was extracted with Et_2O (2×10 ml), dried over $MgSO_4$, filtered, and concentrated in vacuo to give the analytically pure deuterated produces. The yield was determined by GC/mass spectrometry (MS) equipped with capillary column and the D contents of the products were determined by ^1H nuclear magnetic resonance (NMR) spectroscopy on the basis of the integration of the aromatic protons.

Moreover, ^1H NMR and MS data of the products were also added in **Figure S14**.

<u>Ph-≡-Ph</u>

^1H NMR (CD_3OD) δ 2.83 (s, 0.02H), 7.09–7.22 (m, 1H); MS (EI) m/z (%): 186 (14.6) $[M(d_4)]^+$, 185 (2.6) $[M(d_3)]^+$, 184 (1.0) $[M(d_2)]^+$, 183 (0.3) $[M(d_1)]^+$, 182 (0.7) $[M(d_0)]^+$, 93 (100), 92 (13).

<u>Ph-=-COOH</u>

^1H NMR (CD_3OD) δ 2.52-2.57 (m, 0.23H), 2.84-2.89 (m, 0.16H), 7.11–7.24 (m, 1H); MS (EI) m/z (%): 152 (22.2) $[M(d_2)]^+$, 151 (6.6) $[M(d_1)]^+$, 150 (0.9) $[M(d_0)]^+$.

<u>Cl-Ph-COOH</u>

^1H NMR (CD_3OD) δ 7.40–7.46 (m, 1H), 7.54-7.58 (m, 0.06H), 7.99–8.00(m, 1H); MS (EI) m/z (%): 123 (52.3) $[M(d_1)]^+$, 122 (6.8) $[M(d_0)]^+$, 106 (100), 105 (15), 78 (91).

Comment 2: What do the authors mean by the configuration described in the line 213?

Answer 2: In our preliminary experiment, the energetically lowest configuration of the Pd formate species was determined to be trans-AgO-PdH(O)-bridged configuration. This is not directly relevant to the following H-D exchange reaction, but we suppose that such detail information will help the understanding of the readers about the reaction mechanism for the dehydrogenation of formic acid.

Comment 3: The energies calculated in this section for the H/D exchange between the amine and H_2O groups seem too high. The exchange of protons between an ammonium salt and water protons must be a very fast process. Can the authors give an explanation to this?

Answer 3: In this calculation, solvent effect is not considered, and it does not take into account the Grotthus mechanism (so-called bucket-relay mechanism) because it does not contain multiple amines or H_2O molecules. For this reason, we think that the activation energy is overestimated, but there is no problem in relative comparison. If you include multiple amines and H_2O molecules, frequency analysis etc. will be very difficult.

Comment 4: In these calculations the energy of the H_2 formation process through the coupling of the hydride of the metallic nanoparticle and the proton centered in the amine has not been estimated. This information would be of interest to know which the limiting step of the process is.

Answer 4: The activation energies for the release of hydrogen from **5** or **6** and **8** or **9** were determined to be 52.7 and 13.0 kJ/mol, respectively, indicating that rate-determining step is the H-D exchange reaction at the basic amine site with D_2O. These results also suggest that the release of the hydrogen isotope compounds from weakly basic amine is lower than that from strongly basic amine, which explain the higher TOF obtained from PdAg/SA-5 as compared to that for PdAg/SA-1, as shown in **Figure 3**.

In order to clarified these results, the following sentences were added to the text.

(Page 8-9) the preliminary DFT calculations determined the E_a for the release of hydrogen from **5** or **6** and **8** or **9** were 52.7 and 13.0 kJ/mol, respectively, indicating rate-determining step is the H-D exchange reaction at the basic amine site with D_2O. These results also suggest that hydrogen release is accelerated in the presence of weakly basic amines, which accounts for the higher TOF obtained from PdAg/SA-5 as compared to that for PdAg/SA-1, as shown in **Figure 3**.

Comment 5: Since the quantum tunneling mechanism is predominantly manifested in the direct H-D exchange on the surface of metallic nanoparticles, how can the observed experimental effect of the basicity of the amine in the HD/D_2 ratio be understood? This apparent discrepancy between theory and experimental observations must be clarified in the text.

Answer 5: We would like to thank his/her critical comment.

The tunneling probability depends to different degrees on the mass of the moving particle, the barrier height, and the barrier width. Especially, the barrier width mainly determines the probability of tunneling in chemical reactions: the narrower a barrier is, the more likely tunneling is. As shown in **Figure S19**, our calculation results reveal that the T_c correlates well with the absolute value ν_{Im} of the imaginary frequency at the transition state, not the activation energy ΔE_{ZP}. The absolute value ν_{Im} is the curvature at the vicinity of the transition state in the reaction coordinate direction, which significantly influence on the barrier width: the larger a value ν_{Im} is, the narrower a barrier is.

The representative reaction paths used to assess the tunneling effect, such as direct H/D exchange over the PdAg surface, direct H/D exchange on an amine group, and H/D exchange over PdAg in the vicinity of an amine group, proceed at the same time. We think that the T_c of the reaction with highest T_c can be experimentally observed. On the other hand, the lower T_c in path II-V suggests that the H-D exchange at the basic amine cites are largely dominated by classical transitions, thus the effect of basicity of the amine in the HD/D_2 ratio can be experimentally appeared. The crucial point of this study is that our theoretical calculation as well as experimental results provide important new insight into the contribution of tunneling effect in the heterogeneously-catalyzed dehydrogenation of FA. The further detail study is now ongoing in our laboratory.

In order to clarify the above discussions, **Figure S19** and the following sentences were added.

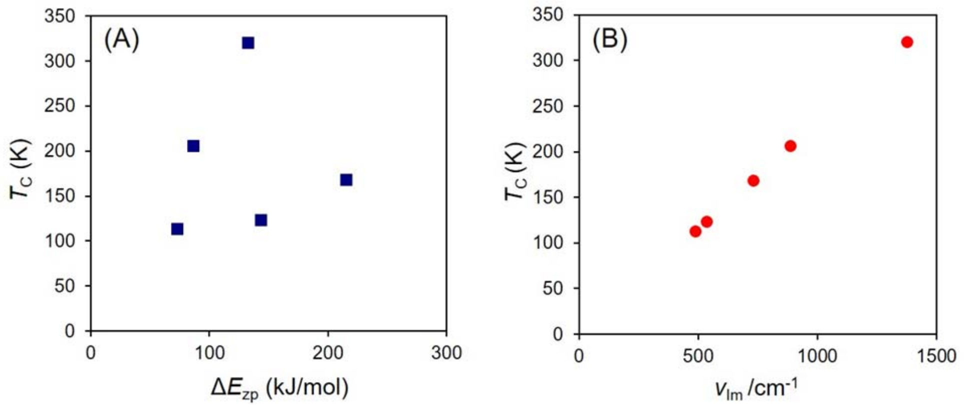

Figure S19. Correlation between tunneling crossover temperature (T_c) and (A) activation barrier (ΔE_{zp}) and (B) frequency (ν_{Im}).

(Page 11-12) The tunneling probability depends to different degrees on the mass of the moving particle, the barrier height, and the barrier width [43]. Especially, the barrier width mainly determines the probability of tunneling in chemical reactions: the narrower a barrier is, the more likely tunneling is [42]. As shown in **Figure S19**, our calculation results reveal that the T_c correlates well with ν_{Im}, not ΔE_{ZP}. The absolute value ν_{Im} is the curvature at the vicinity of the transition state in the reaction coordinate direction, which significantly influence on the barrier width: the larger a value ν_{Im} is, the narrower a barrier is. The representative reaction paths used to assess the tunneling effect proceed at the same time, thus, the T_c of the reaction with highest T_c can be experimentally observed. The lower T_c in path II-V suggests that the H-D exchange at the basic amine cites are largely dominated by classical transitions, thus the effect of basicity of the amine in the HD/D_2 ratio can be experimentally appeared.

[42] Meisner, J. & Kästner, J. Atom tunneling in chemistry. *Angew. Chem. Int. Ed.* **55**, 5400-5413 (2016).

[43] Schreiner, P. R. Tunneling control of chemical reactions: The third reactivity paradigm. *J. Am. Chem. Soc.* **139**, 15276-15283 (2017).

Answer to the comments by Reviewer #3

Comment 1: For XANES spectra and FT-EXAFS spectra in Figure S5 and S6, why chose PdAg/SA-5 and PdAg/SA-1 as the example? Does two of them have any representation?

Answer 1: PdAg/SA-1 modified with strongly basic −NEt_2 groups predominantly evolved HD, while PdAg/SA-5 modified with weakly basic −$PhNH_2$ groups promote the preferential formation of D_2. Thus, we performed the XAFS characterization by using these two representative samples with completely different characteristics.

Comment 2: Is this sentence "It should also be noted that surface amine functional groups were present at the peripheries of the loaded PdAg NPs to alter their electronic state" based on experiments or articles?

Answer 2: The Pd 3d peaks in the XPS spectra produced by the PdAg/SA-*x* are slightly shifted to higher binding energies compared to those for the unmodified sample (**Figure S7A**). A similar shift

of the Ag 3d peaks is also evident after modification (**Figure S7B**). Such experimental results based on XPS analysis suggest that the surface amine functional groups were present at the peripheries of the loaded PdAg NPs to alter their electronic state. The alternation of the electronic state of the supported nanoparticles by the organic modifier of the support materials have also been reported previously.

To clarify the above results, the following sentences were modified, and the relevant reference was added.

Old: Additionally, the Pd 3d peaks in the XPS spectra produced by the PdAg/SA-*x* are slightly shifted to higher binding energies compared to those for the unmodified sample (**Figure S7A**). A similar shift of the Ag 3d peaks is also evident after modification (**Figure S7B**). It should also be noted that surface amine functional groups were present at the peripheries of the loaded PdAg NPs to alter their electronic state.

New: Additionally, the Pd 3d peaks in the XPS spectra produced by the PdAg/SA-*x* are slightly shifted to higher binding energies compared to those for the unmodified sample (**Figure S7A**). A similar shift of the Ag 3d peaks in the XPS spectra is also evident after modification (**Figure S7B**). Our experimental results suggest that surface amine functional groups were present at the peripheries of the loaded PdAg NPs to alter their electronic state. The alternation of the electronic state of the supported nanoparticles by the organic modifier of the support materials have also been reported previously.[33] **(Page 5)**

33 Masuda, S., Mori, K., Futamura, Y. & Yamashita, H. PdAg nanoparticles supported on functionalized mesoporous carbon: Promotional effect of surface amine groups in reversible hydrogen delivery/storage mediated by formic acid/CO_2. *ACS Catal.* **8**, 2277-2285 (2018).

Comment 3: A written error of "TON" in line 197 should be corrected to "TOF".

Answer 3: We would like to thank his/her comment. The "TON" was changed to "TOF". (page 7)

Comment 4: To prove that the selectivity for the production of hydrogen isotope compounds is not governed by the inherent ability of the metal NPs themselves, a bare SA-5 substrate should be tested for the selectivity. Otherwise, the results can only suggest that the selectivity is irrelevant to the interaction between bimetals.

Answer 4: The use of bare SA-5 without PdAg nanoparticles does not show any activity for the dehydrogenation of FA, as deduced from reaction mechanism in **Figure 7**. For that reason, we

investigated the hydrogen isotope selectivity for the dehydrogenation of FA using a series of Pd-based alloy nanoparticles supported on SA-5, as shown in **Figure 6**.

To clarify this, he following sentence was added in the text.

(page 5-6) Utilizing the prepared catalysts having different amine functional groups, the dehydrogenation reaction was conducted in D_2O solutions containing 5 M HCOOH:HCOONa (9:1) at 343 K for 30 min. The selectivities for the resulting hydrogen isotope compounds (HD and D_2) are summarized in **Figure 3**, together with the turnover frequency (TOF) values based on the amount of Pd. No H_2 formation was observed, and the molar ratios of total hydrogen isotope compounds to CO_2 generated during the course of the reaction were close to 1 for all samples. The use of bare SA-5 without PdAg NPs does not show any activity.

Comment 5: In Figure 5, PdAg/S also should be tested for the relation between the basicity and selectivity to prove the point that hat the basicity of the amine functional groups plays a crucial role in determining selectivity.

Answer 5: PdAg/S without the amine modification gave 58% D_2 and 48% HD selectivity, as shown in **Figure 3**. The basicity of a series of the SA-*x* was determined from the adsorption energy (E_{ad}) of formic acid based on the DFT calculations, since the adsorption involves formation of an acid–base pair via N–H···O hydrogen bonding. However, the determination of the surface basicity of the pristine mesoporous silica by same method is difficult, because the surface property of the PdAg/S, which expose Si-OH groups, is quite different from that of the PdAg/SA-*x* modified amine functional groups. For these reasons, we exclude the result of PdAg/S from the plot showing the correlations between HD selectivity and (A) basicity of the amine groups determined by the adsorption energy (E_{ad}) of FA in **Figure 5A**.

REVIEWERS' COMMENTS:

Reviewer #1 (Remarks to the Author):

In the revised manuscript, the authors carefully addressed the comments with additional experiments. Therefore, I recommend the acceptance of this manuscript.

Reviewer #2 (Remarks to the Author):

Nature Communications manuscript NCOMMS-19-17661A
After reading this work it is clear that the criticisms raised by the authors have been adequately addressed. Considering my comments, I consider that the answers, clarifications and explanations given by the authors are correct. Likewise, corrections and additional comments introduced in the text improve the work. Therefore, I consider that this draft could be accepted in Nature Communications in its current form.
